# Loss-of-function variants in human *C12orf40* cause male infertility by blocking meiotic progression

**DOI:** 10.1038/s41421-023-00580-w

**Published:** 2023-08-22

**Authors:** Chaofeng Tu, Junfei Wen, Weili Wang, Qifan Zhu, Ying Chen, Jianglu Cheng, Zeye Li, Lanlan Meng, Yong Li, Wenbin He, Chen Tan, Chunbo Xie, Shao-Mei Fu, Juan Du, Guangxiu Lu, Ge Lin, Lan-Tao Gou, Yue-Qiu Tan

**Affiliations:** 1 Institute of Reproductive and Stem Cell Engineering, NHC Key Laboratory of Human Stem Cell and Reproductive Engineering, School of Basic Medical Sciences, Central South University, Changsha, Hunan China; 2 State Key Laboratory of Molecular Biology, Shanghai Key Laboratory of Molecular Andrology, Shanghai Institute of Biochemistry and Cell Biology, Center for Excellence in Molecular Cell Science, Chinese Academy of Sciences, Shanghai, China; 3 University of Chinese Academy of Sciences, Beijing, China; 4 Chongqing Key Laboratory of Maternal and Fetal Medicine, Chongqing Medical University, Chongqing, China; 5 Clinical Research Center for Reproduction and Genetics in Hunan Province, Reproductive and Genetic Hospital of CITIC-Xiangya, Changsha, Hunan China; 6 Department of Breast Surgeon, The Obstetrics & Gynecology Hospital of Fudan University, Shanghai, China; 7 College of Life Science, Hunan Normal University, Changsha, Hunan China

**Keywords:** DNA recombination, Mechanisms of disease

Dear Editor,

Non-obstructive azoospermia (NOA) stands for the most severe form of male infertility affecting ~1% male population^1^. Clinically, the connections between various gene variants and sterility have been widely observed in NOA patients^2,3^. However, it remains poorly understood which of them are authentic disease-causing genes and how these gene mutations lead to male infertility.

To explore the genetic causes of NOA, we carried out a screening for potential variants in a cohort of 279 NOA-affected individuals by whole-exome sequencing^4^, and identified 4 patients carrying 2 homozygous variants in an unprecedented gene *C12orf40* (NM_001031748.4) from 3 unrelated Chinese families (Fig. 1a; Supplementary Fig. S1 and Table S1), which are rare or even absent in public databases (Supplementary Table S1). Sanger sequencing validated that the probands T00166:II-1 and Y9770:II-1 harbor the frameshift variant M1: c.232_233insTT (p.M78Ifs*2), while T00579:II-1 and II-2 have splice variant M2 (c.1286+1G > A) (Fig. 1a; Supplementary Fig. S2). Histopathological analysis of the patient’s testicular biopsy revealed the presence of spermatocytes but lack of condensed spermatids in seminiferous tubules (Fig. 1b), which is further confirmed by H1t and PNA staining (Fig. 1c; Supplementary Fig. S3). All 4 patients had normal sex hormone levels and no other reproductive abnormities (Supplementary Table S2). These results suggested the homozygous loss-of-function variants in *C12orf40* as a potential cause of meiotic arrest and male infertility.

**Fig. 1:**
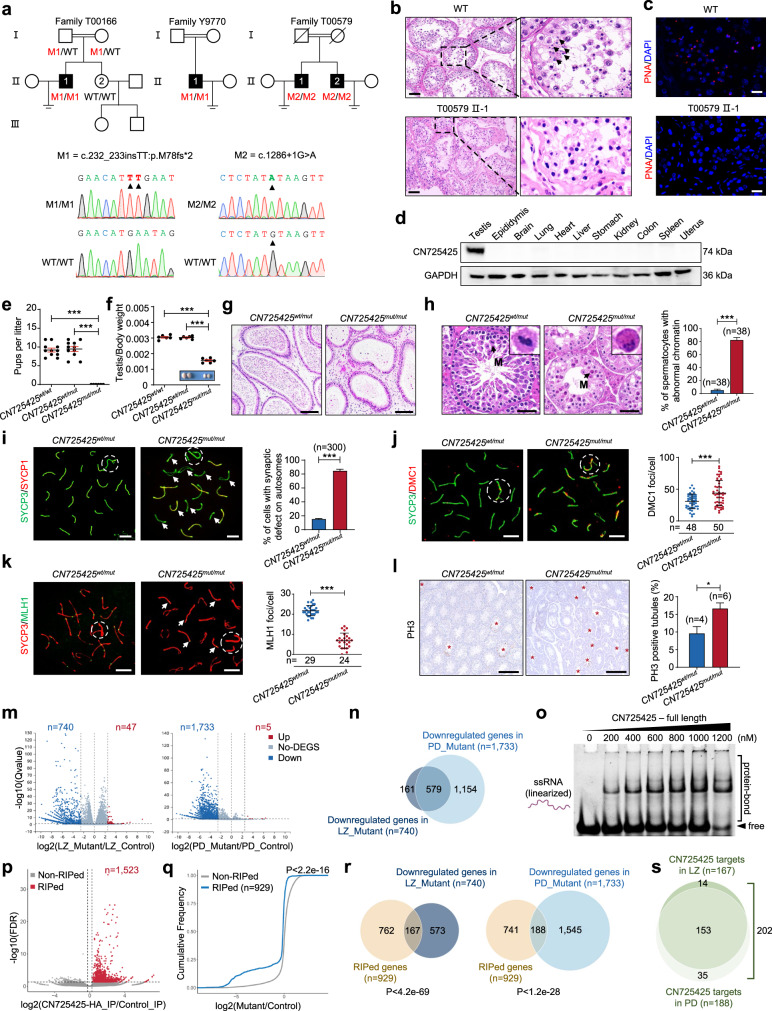
*C12orf40* variants identified in NOA patients cause meiosis arrest and male infertility by destabilizing mRNAs required for germ cell development. **a** Homozygous variants M1 and M2 identified in 3 unrelated Chinese families. The variant positions are indicated by arrow heads. **b** Hematoxylin & eosin staining (H&E) staining of testicular biopsy sections from wild-type (WT) and *C12orf40*-mutant individual (T00579:II-1). The arrow heads indicate the condensed spermatids. Scale bars, 50 μm. **c** Immunostaining of testicular biopsy sections from WT and *C12orf40*-mutant individual (T00579:II-1) for PNA (haploid spermatid marker, red) with nuclei counterstained by DAPI (blue). Scale bars, 20 μm. **d** Western blotting of CN725425 in indicated mouse tissues. GAPDH as internal control. **e** Litter size of adult males after mating with WT females (*n* = 6 per genotype). Error bar, mean ± SEM. ***P < 0.001. **f** The representative image of testes and the ratios of testis/body weight from males at 2-month-old (*n* = 3 per genotype). Error bar, mean ± SEM. ***P < 0.001. **g** H&E staining of cauda epididymis sections from adult mice. Scale bars, 50 µm. **h** Left, H&E staining of testis sections from adult mice, with zoomed image of spermatocytes at first MMI, as indicated by arrows. Scale bars, 50 µm. Right, Quantification of MMI spermatocytes with abnormally condensed chromatin in testes. **i**–**k** Left, immunostaining of pachytene spermatocyte spreads derived from testes for SYCP1 (**i**), DMC1 (**j**), and MLH1 (**k**, red) with SYCP3 (green). The arrows indicate synaptic (**i**) or crossover formation (**k**) defects. The circles indicate sex chromosomes. Scale bars, 10 µm. Right, quantification of synaptic defects on autosomes (**i**), DMC1 foci (**j**), or MLH1 foci (**k**) in pachytene spermatocytes. ***P < 0.001. **l** Left, immumohistochemical analysis of p-ser10-H3 (PH3) of paraffin sections from adult testes. Right, the percentage of tubules containing metaphase I/II spermatocytes was quantified. Scale bars, 10 μm. *P < 0.05. **m** DEG analysis between mutant and WT spermatocytes at LZ/PD stage. **n** Venn diagram showing shared downregulated genes between mutant LZ and PD spermatocytes. **o** EMSA using CN725425 and ssRNA substrate. **p** Volcano plot showing differentially enriched sites by RIP-seq using CN725425-HA knock-in and WT mouse testes. Sites with enriched HA signal (red dot) were considered as RIPed sites. **q** Cumulative abundance of differential expression level between mutant and WT spermatocytes of mRNAs in non-RIPed (gray) and CN725425 RIPed (blue) genes. **r** Venn diagram showing shared genes between CN725425-RIPed mRNAs and downregulated genes in LZ/PD spermatocytes. **s** Venn diagram showing CN725425-regulated RNA targets in LZ/PD spermatocytes.

Notably, *C12orf40* (mouse ortholog CN725425) is dominantly restricted to vertebrate testis and its protein sequence is highly conserved (Fig. 1d; Supplementary Figs. S4 and S5). Immunostaining and single-cell RNA-seq analysis further indicated that *C12orf40/CN725425* appears in spermatogonia and is enriched in spermatocytes, while it is absent in testicular somatic cells (Supplementary Figs. S6 and S7). To demonstrate whether loss-of-function *C12orf40* variants play a causative role in male infertility, we generated a *CN725425* mutant mouse (Supplementary Fig. S8), mimicking the M1 variant. Strikingly, the mutant males were completely sterile (Fig. 1e), with smaller testes and hardly observed sperm in epididymis (Fig. 1f, g) due to the absence of haploid spermatids and dramatically increased spermatocytes with abnormal chromatin morphology (Fig. 1h; Supplementary Fig. S9). Collectively, the recapitulated NOA phenotype in the mutant mouse suggests that *C12orf40* mutations identified in patients are disease drivers.

We next inspected the key biological events occurred during the meiotic prophase I in *CN725425* mutant testes. By immunostaining of γH2AX and RPA2, we found comparable signal patterns and intensities in both mutant and control spermatocytes at leptotene/zygotene ((LZ) stage (Supplementary Fig. S10a, b), suggesting CN725425 is not required for DNA double-strand breaks (DSBs) formation. Then we examined the staining pattern of SYCP1, and found ~85% of *CN725425* mutant pachytene spermatocytes with incomplete synapsis especially at the far-end of centromere region, and ~64% with detached and unsynapsed sex chromosomes (Fig. 1i; Supplementary Fig. S11). We further checked the recombinases and found much more foci of DMC1, RAD51, and RPA2 retained on autosomes in mutant pachytene spermatocytes (Fig. 1j; Supplementary Fig. S10). These results indicated unaffected loading of RPA2, DMC1 and RAD51 to meiotic DSB ends but impaired elimination of them, suggesting a disrupted programmed DSB repair which was further evidenced by the retained γH2AX signals on autosomal axes (Supplementary Fig. S10a) and largely reduced crossover formation (Fig. 1k) in *CN725425* mutant pachytene spermatocytes.

Then we determined the stage of meiosis arrest in mutant mice. Little alterations were found in ratio of spermatocytes at each stage during meiosis prophase I, but the number of bivalent in meiotic metaphase I (MMI) spermatocytes was dramatically reduced (Supplementary Fig. S12a, b). PH3 staining also indicated significant elevation of seminiferous tubules containing MMI spermatocytes in mutant testis (Fig. 1l), with increased apoptosis (Supplementary Fig. S12c). Collectively, these results suggested MMI arrest phenotype, which could probably be explained by the failure of proper segregation of homologous chromosomes caused by the dysregulation all the way from homologous recombination (HR) to programmed DSB repair to crossover formation upon *CN725425* deficiency.

To explore the global impact by CN725425 depletion, we analyzed transcriptome of spermatocytes during LZ-to-Pachytene/Diplotene (PD) transition. We first isolated LZ and PD spermatocytes^5^ for RNA-seq (Supplementary Fig. S13). Unexpectedly, the majority of differentially expressed genes (DEGs) in response to CN725425 depletion were downregulated in LZ/PD spermatocytes (Fig. 1m, n), and most of downregulated genes were enriched in germ cell development/spermiogenesis-related GO categories (Supplementary Figs. S14 and S15). These analyses suggested a hypothesis that CN725425 facilitates meiotic progression by positively regulating a group of key developmental genes. If it is true, there would be two possibilities: (1) CN725425 functions as transcription factor (TF) in activating, (2) or RNA binding protein (RBP) in stabilizing these genes during meiosis, given that CN725425 is predicted as a nucleic acid binding protein (Supplementary Fig. S16). However, by distinguishing the transcriptome change during LZ-to-PD into three clusters, we found, upon CN725425 deletion, the decline of genes (cluster I) was strengthened and the stable genes (cluster II) became degraded, while cluster III were almost not affected (Supplementary Fig. S17). This analysis suggested that the most likely role of CN725425 is stabilizing mRNA as RBP rather than activating genes as TF, at least during LZ-to-PD transition.

To demonstrate this, we purified the recombinant CN725425 protein in bacteria and determined its ability of nucleic acid binding (Supplementary Fig. S18). As prediction, electrophoretic mobility shift assay (EMSA) indicated that CN725425 but not mutant protein possesses significant RNA binding capacity in RNA structure independent manner (Fig. 1o; Supplementary Fig. S18). To test this in vivo, we generated HA-tagged CN725425 knock-in mouse to overcome the lack of antibody for pull-down assay (Supplementary Fig. S19). We performed anti-HA RNA immunoprecipitation sequencing (RIP-seq) with testis lysates from CN725425-HA and control mice at age of 18dpp (Supplementary Fig. S20a, b), a stage that males possess spermatogonia and spermatocytes but lack spermatids in testis. Indeed, a total of 1523 sites of transcripts were co-immunoprecipitated with CN725425-HA (Fig. 1p), in which ~78% of sites located on protein-coding genes (Supplementary Fig. S20c, d). Together with EMSA, this result demonstrated CN725425 as a RBP.

Therefore, we hypothesized that CN725425 functions as RBP to stabilize mRNAs that have been transcribed but need to be protected for translational activation in later stage, to adapt to the global transcriptional silencing^6,7^ and the programmed wave of mRNA degradation^8–10^ around LZ-to-PD transition. Interestingly, we observed that the co-immunoprecipitated transcripts are preferentially downregulated upon CN725425 depletion (Fig. 1q; Supplementary Fig. S21a, b), suggesting the stabilizing role of CN725425 on its binding RNAs. Although 994 gene transcripts were identified as CN725425-HA associated, we speculated that only part of them could be directly regulated because of unavoidable indirect bindings. To determine the regulatory targets, we compared these RIPed mRNAs with DEGs upon CN725425 depletion, 167 and 188 overlapped genes were found in downregulated but not upregulated genes in mutant LZ and PD spermatocytes, respectively (Fig. 1r; Supplementary Fig. S21c, d). We thus assumed the total 202 mRNAs as CN725425’s direct downstream targets (Fig. 1s). Further analysis showed that they were significantly enriched in general germ cell development/spermiogenesis-related GO categories, with strong binding preference by CN725425 at 3’UTR regions (Supplementary Fig. S22). These results further supported the model that CN725425 binds and protects a population of germ cell development and even spermiogenesis-related transcripts from the wave of mRNA degradation around LZ-to-PD transition, in order to facilitate meiotic progression.

Interestingly, we found that CN725425 but not mutant protein associates to double-strand DNA molecules as well (Supplementary Fig. S23). Therefore, we could not completely rule out that CN725425 simultaneously has other potential function(s) to contribute meiosis through its DNA binding behavior despite of its verified major role in RNA metabolic regulation.

In summary, we identified 2 homozygous *C12orf40* mutations in 4 NOA patients from 3 unrelated families, and by modeling such loss-of-function variant in mouse, we demonstrated that lack of *C12orf40*/*CN725425* causes meiotic arrest and male infertility, with extensive defects in synapsis and crossover formation during meiotic recombination and DSB repair. Importantly, we further established that CN725425 functions as a RBP and orchestrates meiosis progression by implicating in stabilization of mRNAs required for germ cell development. Thus, based on the evidences presented here, we report *C12orf40* as a new causative gene in human infertility.

### Supplementary information

Supplementary file

## Data Availability

The RNA-seq and RIP-seq data generated under this study can be accessed from SRA: PRJNA866911. Custom code is available upon request.
