# Supplementary material for: Loss-of-function variants in human C12orf40 cause male infertility by blocking meiotic progression
Source: Cell Discov. 2023 Aug 22;9:87. doi: 10.1038/s41421-023-00580-w (PMC10442395; doi:10.1038/s41421-023-00580-w)
Supplement: Supplementary file 1 — Supplementary file [file 41421_2023_580_MOESM1_ESM.pdf]

Supplementary Information for

Loss-of-function Variants in Human *C12orf40* Cause Male Infertility by Blocking Meiotic Progression

Chaofeng Tu[1,5]†, Junfei Wen[2,3]†, Weili Wang[1,5,7]†, Qifan Zhu[2,3]†, Ying Chen[2,4], Jianglu Cheng[2,3], Zeye Li[2,3], Lanlan Meng[1,5], Yong Li[1], Wenbin He[5], Chen Tan[1], Chunbo Xie[1,5], Shao-Mei Fu[6], Juan Du[1,5], Guangxiu Lu[5], Ge Lin[1,5]*, Lan-Tao Gou[2,3]*, and Yue-Qiu Tan[1,5,7]*

[1]Institute of Reproductive and Stem Cell Engineering, NHC Key Laboratory of Human Stem Cell and Reproductive Engineering, School of Basic Medical Sciences, Central South University, Changsha 410078, China

[2]State Key Laboratory of Molecular Biology, Shanghai Key Laboratory of Molecular Andrology, Shanghai Institute of Biochemistry and Cell Biology, Center for Excellence in Molecular Cell Science, Chinese Academy of Sciences, Shanghai 200031, China

[3]University of Chinese Academy of Sciences, Beijing 100049, China

[4]Chongqing Key Laboratory of Maternal and Fetal Medicine, Chongqing Medical University, Chongqing 400016, China

[5]Clinical Research Center for Reproduction and Genetics in Hunan Province, Reproductive and Genetic Hospital of CITIC-Xiangya, Changsha 410008, China

[6]Department of Breast Surgeon, The Obstetrics & Gynecology Hospital of Fudan University, Shanghai 200011, China

[7]College of Life Science, Hunan Normal University, Changsha, Hunan 410081, China

† These authors contributed equally

* Correspondence: linggf36@hotmail.com (G.L.), goulantao@sibcb.ac.cn (L.-T.G.), tanyueqiu@csu.edu.cn (Y-Q.T.)

This file includes:

Materials and Methods

Supplementary References

Supplementary Figures S1 to S23

Supplementary Tables S1 to S4

Materials and Methods

Human subjects

A cohort of 279 NOA-affected Chinese men were recruited from the Reproductive and Genetic Hospital of CITIC-Xiangya (Changsha, China). Semen analysis of the participating individuals were performed at least twice and revealed no spermatozoa with normal volume according to the World Health Organization (WHO, 2010) guidelines [1]. The reproductive hormones in the serum of NOA-affected individuals were determined in the local laboratory. All individuals have normal somatic chromosomal karyotypes; no abnormalities were detected for azoospermia factor microdeletion on Y chromosome. Other causes of infertility, such as drugs and exposure to gonadotoxic factors, were also excluded. The present study was approved by the Institutional Ethics Committees of the Central South University and Reproductive Genetic Hospital of CITIC-Xiangya (LL-SC-2017). All methods were performed in accordance with the approved guidelines. Written informed consent was signed by all participating individuals at the beginning of the study.

Whole-exome sequencing (WES), bioinformatics analysis and Sanger sequencing

Genomic DNA extraction, subsequent WES and data analysis for the NOA-affected individuals were carried out by Beijing Genome Institute (BGI) in Shenzhen as described previously [2]. All available individuals were sequenced to at least a mean depth of 100×. The most promising candidate variants were identified using the criteria as we previously described [2]. In brief, a candidate variant was preferentially considered if it was 1) reported to have a frequency below 1% in public databases, including 1000 Genomes Project and gnomAD (v2.1.1); 2) homozygous variants were considered with priority; 3) predicted to be deleterious variants using different tools (Polyphen-2, MutationTaster, SIFT, and Combined Annotation Dependent Depletion); and 4) associated with phenotype, including high expression in testis or the existence of a gene knockout mouse model with infertile phenotype. Meanwhile, homozygosity mapping was performed using HomozygosityMapper, and homozygous variants which located in homozygous regions greater than 5.0 Mb were considered with priority.

The variants (NM_001031748.4: c.232_233insTT and c.1286+1G>A) of *C12orf40* were validated by Sanger sequencing using specific primers listed in Supplementary Table S3. The variant sites in *C12orf40* were examined in patients and their family members by PCR using Ex Taq DNA polymerase (BIO-RAD). All PCR products were sequenced on a 3730XL sequencer (Applied Biosystems, San Diego, Foster City, CA, USA) according to the manufacturer's instructions.

Reverse Transcription PCR (RT-PCR) and RT-NESTED-PCR analysis

Total RNA was isolated from mouse tissues to analyze the expression of *C12orf40* mRNA using Trizol extraction kit (Invitrogen, Carlsbad, CA, USA), and 1 μg RNA was reverse transcribed into cDNA using a goScript reverse transcription system (Promega, Madison, WI, USA). 10 ng of cDNA was used as templates for the subsequent RT-PCR using specific primers listed in Supplementary Table S3, and β-actin was used as an internal control.

For RT-NESTED-PCR analysis, total RNA of blood samples from subject T005792 II -1 and normal control were extracted and reverse transcribed as described above. The first round of RT-PCR was performed using 10 ng of cDNA, and the RT-PCR products were used as templates for the second round of RT-PCR underling the same conditions. Subsequent, the PCR products were analyzed by 2.0% agarose gel electrophoresis to determine the band size, and sequenced on a 3730XL sequencer (Applied Biosystems, San Diego, Foster City, CA, USA).

Mice model generation

All animal procedures were carried out according to the protocols established by the Institutional Animal Care and Use Committee of Central South University (Changsha, China) and the CEMCS Institutional Animal Care and Research Advisory Committee (Shanghai, China).

CN725425 mutant mice were generated by using CRISPR/Cas9-mediated genome editing approach [3]. Briefly, exon 4 (ENSMUST00000190436, c.232_233) of

CN725425 was selected as target site, and two single-guide RNAs (sgRNA-1: CTGGTCATATTCACATGAGTTGG, sgRNA-2: TAGAGGGACTTTCAAATCTCT GG) were designed. Cas9 mRNA and sgRNAs generated by *in vitro* transcription were co-microinjected into the fertilized eggs. Then embryos were transplanted into the oviducts of pseudo-pregnant ICR females. Offspring were genotyped by PCR using toe genomic DNA with the specific primers and identified by Sanger sequencing. The specific primers were listed in Supplementary Table S3. The mice at 8-16 weeks of age were used for fertility testing as we described earlier [2].

CN725425-HA knock-in mice were generated by the Animal Core Facility of CMECS using CRISPR/Cas9-mediated genome editing approach [3]. Briefly, exon 13 (ENSMUST00000190436, c.2173_2174) of *CN725425* was selected as target site, and a single-guide RNA (sgRNA: TCACTCTAACATTTCCAACTAGG) and a homologous recombinant donor (HA-ssODN: CCAAGGGTAAATGAAACACTGCT TCCTAGTTGGAAATGTTAAGCGTAATCTGGTACGTCGTATGGGTAGAGTG AGTTACTTGTAGGTGTTTCCTGCCC) were designed. Cas9 mRNA and sgRNAs generated by *in vitro* transcription, and ssODN synthesized by Azenta Life Sciences were co-microinjected into the fertilized eggs. Then embryos were transplanted into the oviducts of pseudo-pregnant ICR females. Offspring were genotyped by PCR using toe genomic DNA with the specific primers and identified by Sanger sequencing. The specific primers were listed in Supplementary Table S3.

Histological and immunostaining analysis, and TUNEL assay

For histological analysis, testes and epididymis were fixed in 4 % paraformaldehyde or Bouin's solution (Sigma-Aldrich) overnight at 4 °C, and then embedded in paraffin, sectioned, processed, stained with hematoxylin and eosin (H&E), and analyzed for pathologic alterations. Immunostaining on the slides was performed as we described previously [4]. Briefly, the primary antibodies were added to the sections and incubated overnight at 4 °C, followed by incubation with the secondary antibody. The nuclei were stained with DAPI. The immunofluorescence images were captured using an Olympus

IX51 fluorescence microscope (Olympus, Tokyo, Japan). The antibodies used in the study are listed in Supplementary Table S4.

Terminal deoxynucleotidyl transferase-mediated dUTP nick end labeling (TUNEL) assay was performed to detect apoptotic germ cells using the DeadEnd™ Colorimetric TUNEL System (Promega, Madison, WI, USA) according to the manufacturer's instructions. Digital images were photographed using an Olympus IX51 fluorescence microscope (Olympus, Tokyo, Japan).

Spermatocyte spreads and immunostaining

Testicular tissue of control and mutant adult mice were macerated in phosphate-buffered saline (PBS) solution, followed by removal of the tunica albuginea. The seminiferous tubules were separated and transferred to the hypotonic buffer (0.6 mol/L Tris pH 8.2, 500 μL; 0.5 mol/L sucrose, 1 mL; 0.17 mol/L trisodium citrate dihydrate, 1 mL; 0.5 mol/L EDTA, pH 8.0, 100 μL, 0.5 mol/L DTT, 50 μL; 0.1 mol/L PMSF, 100 μL; ddH2O, 7.25 mL) for 30 min. After crushed in 100 mm sucrose buffer (pH 8.2), the cell suspension was gently spread onto slides with fixative buffer (1% PFA and 0.15% Triton X-100), followed by incubation in a moist box for 12 hr at room temperature. Subsequently, the slides were air-dried, washed with PBST three times (PBS containing 1% Triton X-100), and stained for immunofluorescence analysis. The slides were blocked with 5% bovine serum albumin and incubated with the primary antibody listed in Supplementary Table S4 for overnight at 4 °C. Slides were then washed and incubated with Alexa Fluor 488- or 555-conjugated secondary antibodies and observed using the Olympus IX51 fluorescence microscope (Olympus, Tokyo, Japan) and analyzed using the VideoTesT-FISH 2.0 software. Semi-quantitative analysis of the fluorescence signals was conducted using ImageJ (NIH Imaging program).

Metaphase I chromosome spreads

Meiotic preparations were made as previously described [5]. Briefly, single-cell suspensions isolated from seminiferous tubule of control and mutant adult mice

fragments were prepared in 2.2% (w/v) trisodium citrate dihydrate (isotonic solution) and centrifuged for 10 min at 1200 rpm, followed by treatment with 1% (w/v) trisodium citrate dihydrate (hypotonic solution) for 20 min at 37 °C and fixation in Carnoy's solution (25% acetic acid, 75% methanol) at 4 °C. After three washes in fixative, chromosome preparations were made by dropping the cell suspension onto cold slides. Slides were dried and stained with Giemsa. To determine meiotic delay, first meiotic metaphases (MMI) were counted in slide areas in which 500 mid-pachytene nuclei per genotype were counted.

Western Blotting

The proteins of mice testicular tissues were extracted using a RIPA lysis buffer (P0013B, Beyotime Biotechnology) that was supplemented with Protease Inhibitor Cocktail (P1006, Thermo Fisher Scientific), then denatured at 100 °C for 10 min. The denatured proteins were separated by 10% SDS-polyacrylamide gels and transferred to a polyvinylidene difluoride (PVDF) membrane (IPVH00010, Millipore). Membranes were blocked in 5% non-fat milk for 2 hr at room temperature, and incubated overnight at 4°C with the following primary antibodies listed in Supplementary Table S4. Then, the membranes were washed in TBST (Tris-buffered saline with Tween-20) for three times, and incubated with secondary antibodies for 1 hr at room temperature. After washing three times in TBST, the blots were revealed using the ECL Western blotting kit (WP20005, Thermo Fisher Scientific).

Isolation of LZ and PD spermatocytes

Leptotene-zygotene (LZ) and pachytene-diplotene (PD) spermatocytes are isolated according to previously described protocol [6]. Briefly, testes from mice at the age of 25 days were dissected. After removal of tunica albuginea, the testes were incubated in 5 ml of 1 µg/mL Collagenase I (Sigma, SCR103) diluted in PBS at 32 °C with gentle agitation for 15 min. The dispersed seminiferous tubules were further digested in 5 ml of 0.25% Trypsin (Corning, 25-052-CI) and 0.001% DNase I (Sigma, DN25) at 32 °C with gentle agitation for another 15 min. The digested cells were later stained with 10

μg/mL Hoechst 33342 (Sigma, B2261) and 2 μg/mL propidium iodide (PI) (Sigma, 81845) diluted in Dulbecco's Modified Eagle's Medium (DMEM) (Corning, 10-013-CV) with 5% fetal bovine serum (FBS) at 32 °C for 40 min. Finally, cells were sorted using FACSAria SORP (BD Biosciences) according to their fluorescent label with Hoechst 33342/PI staining.

RNA immunoprecipitation

RNA immunoprecipitation (RIP) assay was performed according to previously described protocol [7]. In brief, testes from at the age of 18 days were homogenized in Lysis Buffer (50 mM Tris-HCl (pH 7.4), 1% Triton X-100, 150 mM NaCl, 5 mM EDTA, protease inhibitor cocktail and RNase inhibitor). Cell extracts were incubated with the HA antibody-coupled Protein A/G beads (Thermo, 88802) for 3-4 h. After washing the beads with washing buffer (50 mM Tris-HCl (pH 7.4), 0.1% Triton X-100, 500 mM NaCl, 5 mM EDTA, protease inhibitor cocktail, and RNase inhibitor) for 3 times, total RNAs were isolated with TRIzol for deep sequencing.

Recombinant protein purification

Full length mouse CN725425 cDNA were cloned into pET-28a vector. Recombinant CN725425 protein was overexpressed in E. coli Rosetta (DE3) strain in Lysogeny broth (LB) medium (Oxoid). The cells were grown at 37°C until OD600 reached 0.8 and then induced with 0.2 mM isopropyl β-D-1-thiogalactopyranoside (IPTG, Sangon Biotech) at 16°C for 20 hr. Cell pellets were resuspended in buffer A (20 mM Tris-HCl, pH 8.0, 500 mM NaCl, 5% glycerol, 20 mM imidazole, 1 mM phenylmethylsulfonyl fluoride), lysed by the supersonic cell disrupter, and centrifuged at 18,000 rpm for 1 hr at 4°C. The supernatant containing CN725425 protein was mixed with 1 mL HisSep Ni-NTA Agarose Resin (Yeasen, 20502) for 1 hr at 4°C and loaded to chromatography columns (Bio-Rad, 7321010), pre-equilibrated in buffer A and eluted with buffer A supplemented with 480 mM imidazole. The flow-through fraction was further dialyzed against buffer B (20 mM Tris-HCl, pH 8.0, 300 mM NaCl, 5 mM DTT). M1 mutation of CN725425 were generated based on PCR-based method. The mutants were purified

by the same method as described above. The purity of purified recombinant proteins were examined by Coomassie brilliant blue staining assay.

Electrophoretic mobility shift assay

The purified CN725425 protein and 250 nM ssRNA substrates (structured or linearized in vitro transcribed $tRNA^{iMet}$) or 50 nM dsDNA substrates (PCR amplified 100-nt dsDNA oligos: TACCTCGCTCTGCTAATCCTGTTACCAGTGGCTGCTGCCAGTGGCGATAAG TCGTGTCTTACCGGGTTGGACTCAAGACGATAGTTACCGGATAAGGCGC) were incubated with 1x EMSA buffer (20 mM Tris-Cl, pH 7.4, 150 mM NaCl, 1 mM DTT, 12% glycerol) for 20 min at 37°C, followed by addition of bromophenol blue loading dye and electrophoresis on 6% native polyacrylamide gel for 60 min at 80V on ice. The gels were subsequently stained with SYBR Gold (Invitrogen, S11494) and scanned by Gel Imager (Tanon).

RNA-seq analysis

Processing of RNA-seq data was performed with BGI's Dr.Tom System (https://gtech.bgi.com/bgi/home). In brief, The sequencing data was filtered with SOAPnuke (v1.5.2) [8] and clean reads were aligned to mouse genome GRCm38/mm10 using HISAT2 (v2.0.4) [9] . Clean reads were also mapped to GRCm38/mm10 coding gene set using Bowtie2 (v2.2.5) [10], then RSEM (v1.2.12)[11] was used for estimating the expression level of genes and transcripts. Differential expression analysis was performed using DESeq2(v1.4.5)[12]. GO enrichment analysis was performed by clusterProfiler (v4.2.2)[13] using hypergeometric test with p value adjusted by Bonferroni correction. Venn diagrams and heatmaps were plotted by R package VennDiagram (v1.7.3)[14] and pheatmap (v1.0.12)[15], respectively.

Single-cell RNA-seq analysis

Raw count matrices downloaded from GEO (GSE109033) were imported to Seurat (v4.1.1)[16] and analysed as previously described by Hermann et al. In brief, raw counts

were filtered (cells expressing ≥ 200 detected genes, genes expressed in ≥ 3 cells) log normalized and scaled. Unsupervised cell clustering and tSNE analysis were performed in Seurat. Raw count matrices without testicular somatic cells were imported to Monocle2 (v2.22.0)[17] and used for additional combined t-SNE and unsupervised density peaks clustering. Significantly variable genes among cells were identified and used for dynamic trajectory analysis which ordered cells in pseudotime. Lists of selected genes identified in aforementioned RNA-seq data were plotted across the pseudotime.

RIP-seq Analysis

The RIP-seq data were trimmed with Cutadapt (v1.18) [18]. Clean reads were first aligned to mouse rRNA with Bowtie (v1.2.1.1) [19]. Unmapped reads were then aligned to mouse genome GRCm38/mm10 with gene annotation GENECODE V25 using STAR (v 2.9) [20]. Mapped reads for annotated genes were counted using STAR default settings. Raw read counts were normalized with DEseq2 (v1.38.3) [21] and enriched genes were detected with a double threshold on the log2-fold change (>1) and the correspondent statistical significance (P value <0.05). Piranha (v0.11) was used to identified the binding site from RIP-seq data [22]. HA-enriched binding sites were identified using DiffBind (v3.8.4) (FDR value <0.05) [23]. GO enrichment analysis was performed using clusterProfiler (v4.6.2) [13].

Statistical analysis

Statistical analysis was performed by Student's t-test, one-way ANOVA, Two Samples Wilcoxon Test or hypergeometric distribution using GraphPad PRISM version 5.01 (GraphPad Software, Inc., La Jolla, CA, USA; RRID:SCR_002798). Data are presented as the mean ± standard error of the mean. Differences were considered significant when the p-value < 0.05 (*), 0.01 (**), or 0.001(***).

Supplementary References

1. Cooper, T. G. *et al.* World Health Organization reference values for human semen characteristics. *Hum Reprod Update* **16**, 231-245, (2010).
2. Tan, Y. Q. *et al.* Loss-of-function mutations in TDRD7 lead to a rare novel syndrome combining congenital cataract and nonobstructive azoospermia in humans. *Genet Med* **21**, 1209-1217, (2019).
3. Paquet, D. *et al.* Efficient introduction of specific homozygous and heterozygous mutations using CRISPR/Cas9. *Nature* **533**, 125-129, (2016).
4. Wang, W. *et al.* CFAP65 is required in the acrosome biogenesis and mitochondrial sheath assembly during spermiogenesis. *Hum Mol Genet* **30**, 2240-2254, (2021).
5. Peters, A. H., Plug, A. W., van Vugt, M. J. & de Boer, P. A drying-down technique for the spreading of mammalian meiocytes from the male and female germline. *Chromosome Res* **5**, 66-68, (1997).
6. Gaysinskaya, V., Soh, I. Y., van der Heijden, G. W. & Bortvin, A. Optimized flow cytometry isolation of murine spermatocytes. *Cytometry A* **85**, 556-565, (2014).
7. Gou, L. T. *et al.* Pachytene piRNAs instruct massive mRNA elimination during late spermiogenesis. *Cell Res* **24**, 680-700, (2014).
8. Li, R., Li, Y., Kristiansen, K. & Wang, J. SOAP: short oligonucleotide alignment program. *Bioinformatics* **24**, 713-714, (2008).
9. Kim, D., Langmead, B. & Salzberg, S. L. HISAT: a fast spliced aligner with low memory requirements. *Nat Methods* **12**, 357-360, (2015).
10. Langmead, B. & Salzberg, S. L. Fast gapped-read alignment with Bowtie 2. *Nature Methods* **9**, 357-359, (2012).
11. Li, B. & Dewey, C. N. RSEM: accurate transcript quantification from RNA-Seq data with or without a reference genome. *BMC Bioinformatics* **12**, 323, (2011).
12. Love, M. I., Huber, W. & Anders, S. Moderated estimation of fold change and dispersion for RNA-seq data with DESeq2. *Genome Biology* **15**, 550, (2014).
13. Wu, T. *et al.* clusterProfiler 4.0: A universal enrichment tool for interpreting omics data. *Innovation (Camb)* **2**, 100141, (2021).

14. Chen, H. & Boutros, P. C. VennDiagram: a package for the generation of highly-customizable Venn and Euler diagrams in R. *BMC Bioinformatics* **12**, 35, (2011).

15. Kolde, R. *pheatmap: Pretty Heatmaps*, <https://CRAN.R-project.org/package=pheatmap> (2019).

16. Hao, Y. *et al.* Integrated analysis of multimodal single-cell data. *Cell* **184**, 3573-3587.e3529, (2021).

17. Qiu, X. *et al.* Single-cell mRNA quantification and differential analysis with Census. *Nature Methods* **14**, 309-315, (2017).

18. Kechin, A., Boyarskikh, U., Kel, A. & Filipenko, M. cutPrimers: A New Tool for Accurate Cutting of Primers from Reads of Targeted Next Generation Sequencing. *J Comput Biol* **24**, 1138-1143, (2017).

19. Langmead, B., Trapnell, C., Pop, M. & Salzberg, S. L. Ultrafast and memory-efficient alignment of short DNA sequences to the human genome. *Genome Biol* **10**, R25, (2009).

20. Dobin, A. *et al.* STAR: ultrafast universal RNA-seq aligner. *Bioinformatics* **29**, 15-21, (2013).

21. Love, M. I., Huber, W. & Anders, S. Moderated estimation of fold change and dispersion for RNA-seq data with DESeq2. *Genome Biol* **15**, 550, (2014).

22. Uren, P. J. *et al.* Site identification in high-throughput RNA-protein interaction data. *Bioinformatics* **28**, 3013-3020, (2012).

23. Ross-Innes, C. S. *et al.* Differential oestrogen receptor binding is associated with clinical outcome in breast cancer. *Nature* **481**, 389-393, (2012).

24. Guo, J. *et al.* The adult human testis transcriptional cell atlas. *Cell Res* **28**, 1141-1157, (2018).

25. Ernst, C., Eling, N., Martinez-Jimenez, C. P., Marioni, J. C. & Odom, D. T. Staged developmental mapping and X chromosome transcriptional dynamics during mouse spermatogenesis. *Nat Commun* **10**, 1251, (2019).

26. Hermann, B. P. *et al.* The Mammalian Spermatogenesis Single-Cell Transcriptome, from Spermatogonial Stem Cells to Spermatids. *Cell Rep* **25**, 1650-1667 e1658, (2018).

27. Cozzetto, D., Minneci, F., Currant, H. & Jones, D. T. FFPred 3: feature-based function prediction for all Gene Ontology domains. *Sci Rep* **6**, 31865, (2016).

28. Su, H., Liu, M., Sun, S., Peng, Z. & Yang, J. Improving the prediction of protein-nucleic acids binding residues via multiple sequence profiles and the consensus of complementary methods. *Bioinformatics* **35**, 930-936, (2019).
29. Oldfield, C. J., Peng, Z. & Kurgan, L. Disordered RNA-Binding Region Prediction with DisoRDPbind. *Methods Mol Biol* **2106**, 225-239, (2020).
30. Cooper, T. G. *et al.* World Health Organization reference values for human semen characteristics. *Hum. Reprod. Update* **16**, 231-245, (2010).

Supplementary figures

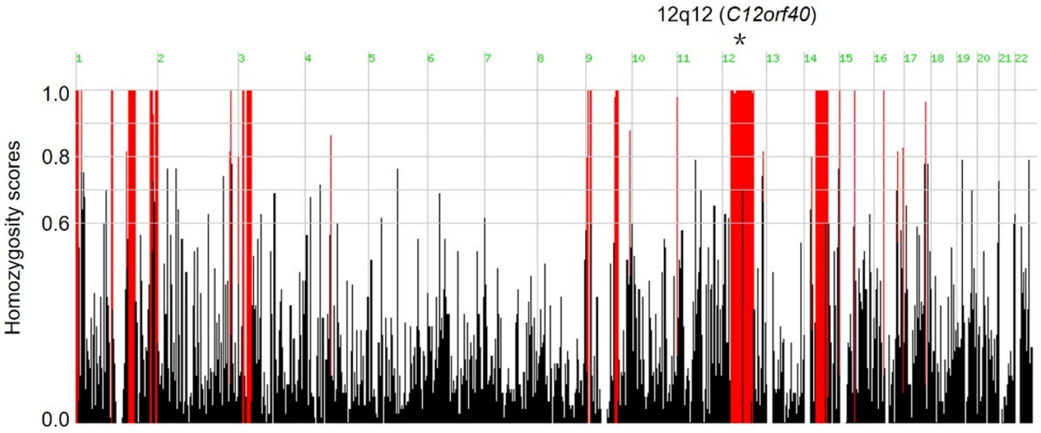

Supplementary Fig. S1 Homozygosity mapping of the affected sibling in family T00166.

Homozygous regions with strong signals are labelled in red. The asterisk indicates the genomic region where *C12orf40* is located.

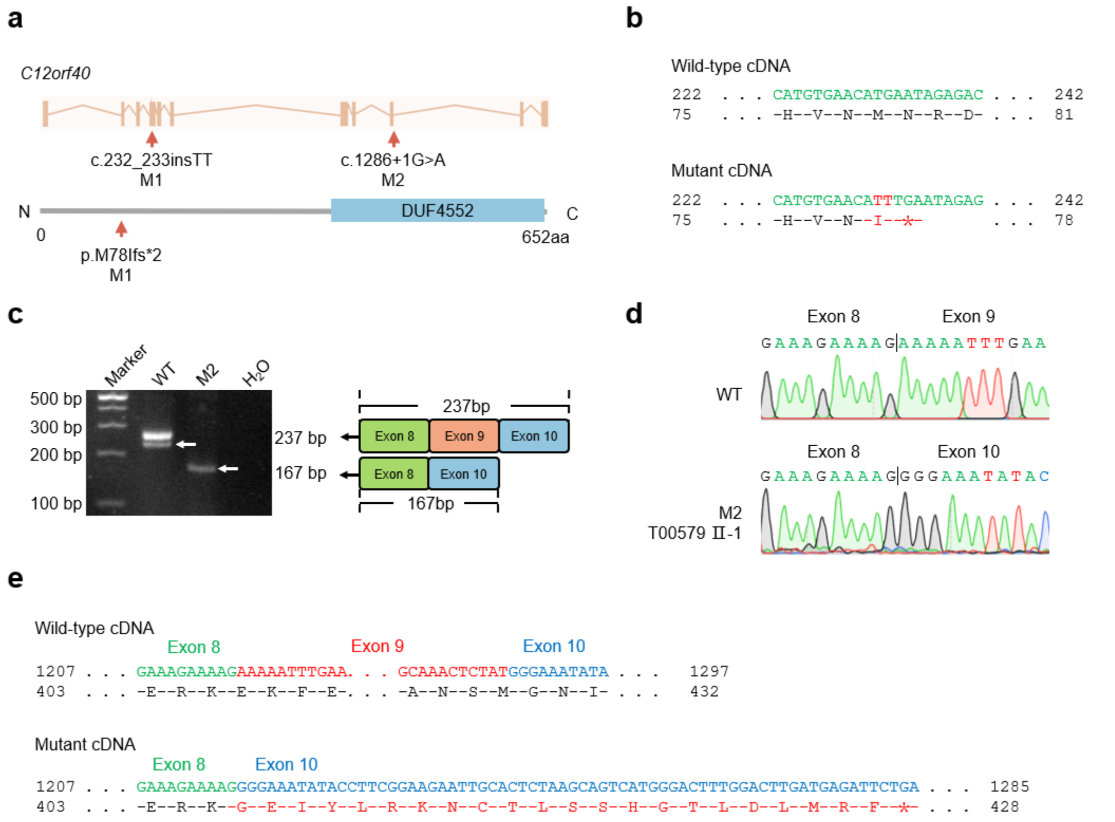

Supplementary Fig. S2 Functional consequences of the *C12orf40* variants identified from NOA patients.

a Schematic map of the variant positions in *C12orf40*. The genomic structure is based on the Ensembl database (GRCh38, transcript ID: ENST00000324616.9). The brown solid squares represent exons, and the lines indicate introns. The only known domain of C12orf40 is the "domain of unknown function" (DUF4552, 425-649aa). **b** Functional consequences of M1 variant (c.232_233insTT). The M1 variant leads to frameshift of C12orf40 protein (p.M78Ifs*2) from the N terminal and consequently a truncated C12ORF40 protein product with 78 amino acids. **c-e** Functional consequences of M2 variant (c.1286+1G>A) on RNA splicing. (**c**) Left: RT-NESTED-PCR analysis of *C12orf40* transcripts spanning exons 8-10 with blood samples from Family T005792. Right: schematic diagram of WT and mutant transcripts (237 bp v.s. 167 bp). (**d**) Chromatogram of *C12orf40* cDNA sequences by RT-NESTED-PCR indicate the skipping of exon 9 caused by M2 variant, giving rise to an out-of-frame fusion of exons 8 and 10. (**e**) The M2 variant leads to a premature termination codon in exon 10, and resulting 428aa truncated protein product.

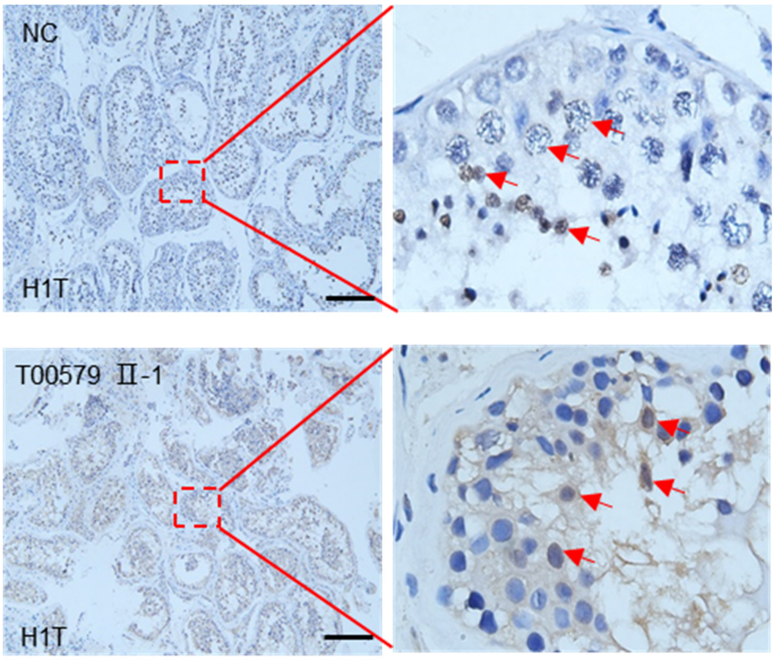

Supplementary Fig. S3 Impaired spermatogenesis of *C12orf40* mutant NOA patient.

Immunohistological analysis of testicular biopsy sections from wild-type and *C12orf40*-mutant individual (T00579 II-1) for H1t (a testis specific histone variant that became detectable from mid-pachytene stage, brown). The red arrows indicate the H1t-positive germ cells. Scale bar, 100 μm.

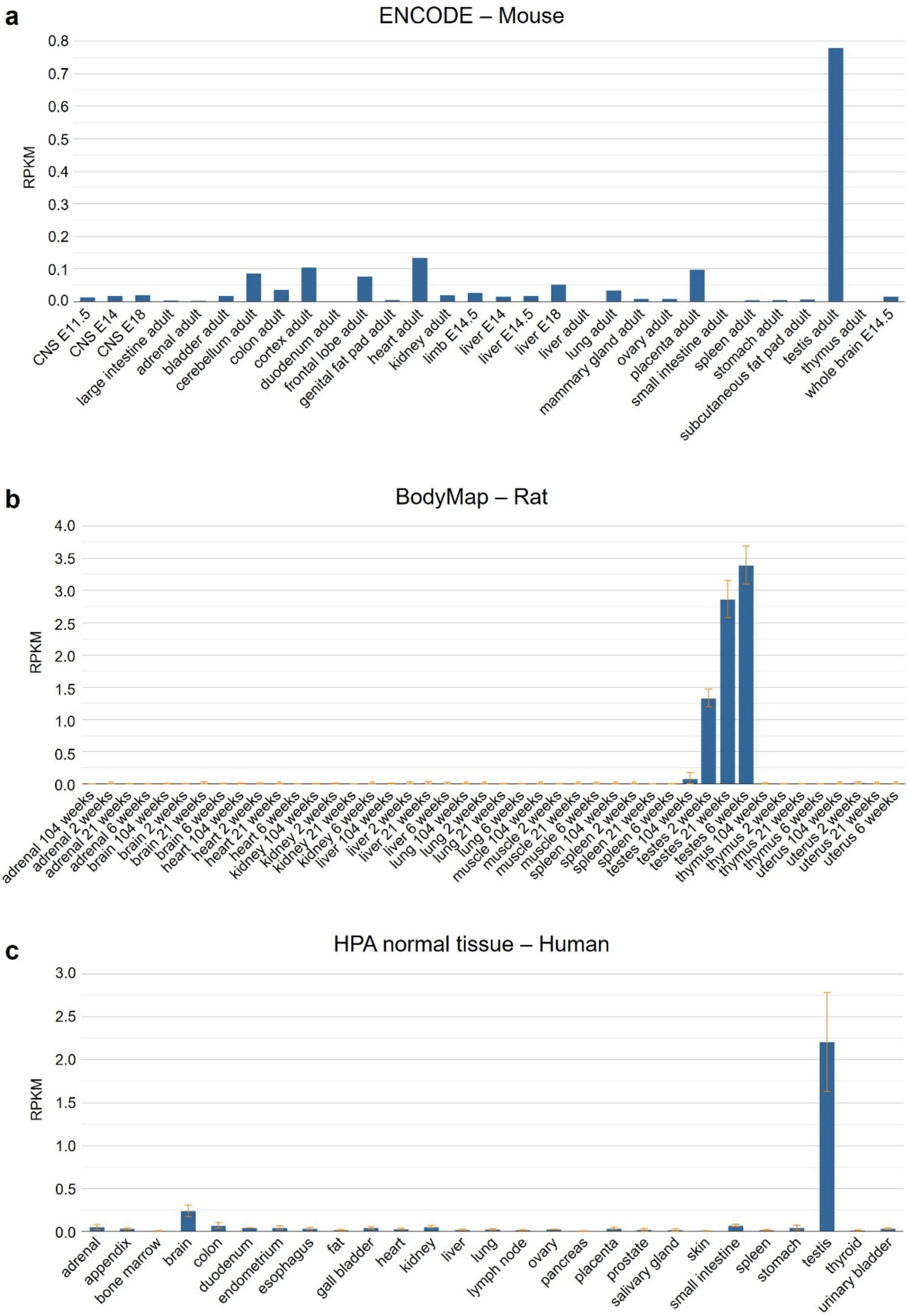

Supplementary Fig. S4 Testis-specific expression of *C12orf40* in vertebrates.

a-**c** Tissue expression profile of *C12orf40* in mouse (**a**), Rat (**b**), and human (**c**), data from public resources.

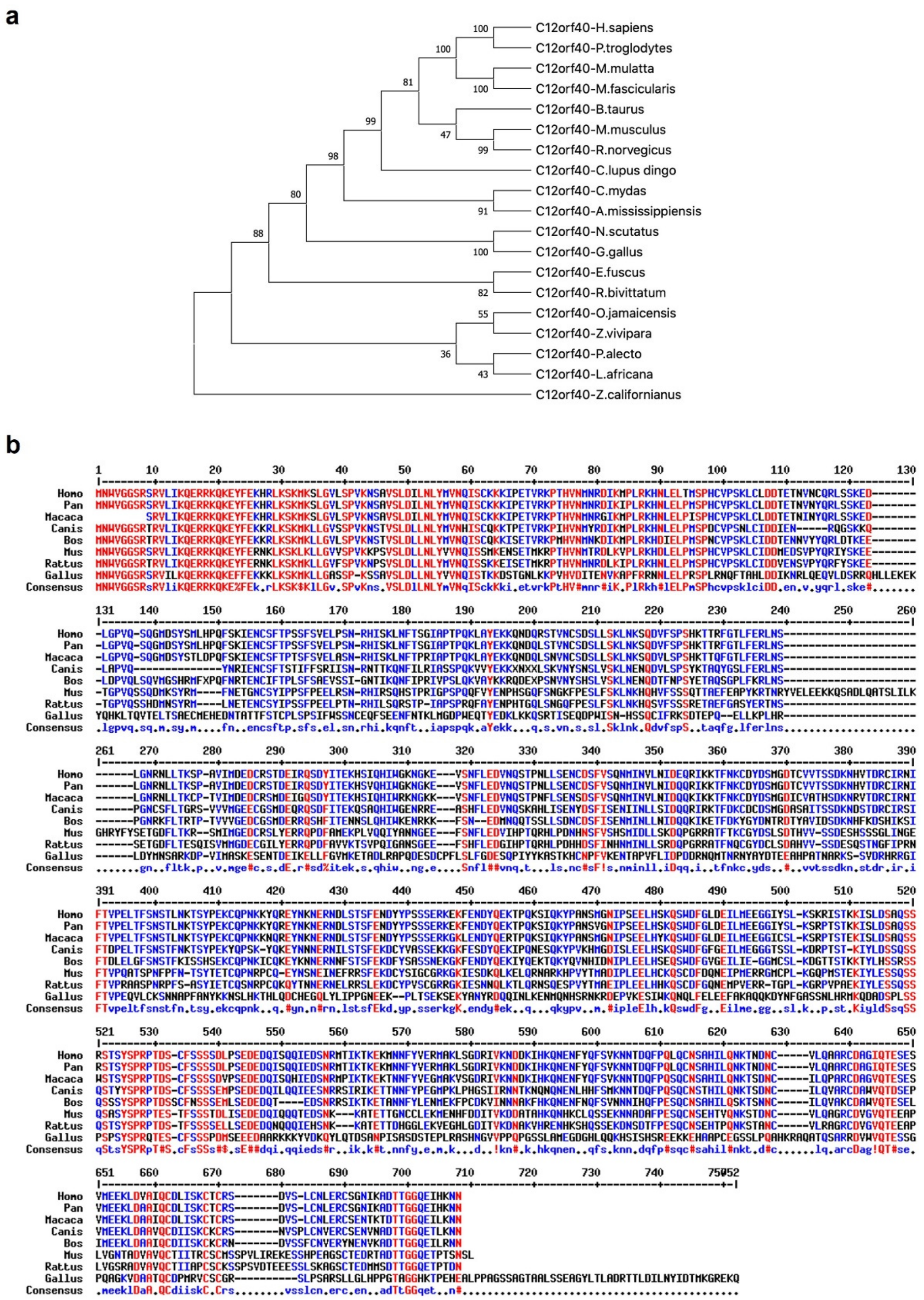

Supplementary Fig. S5 Evolutionary conservation of human C12orf40 protein.

a Evolutionary tree of human C12orf40 homologous proteins in vertebrates. **b** Conserved protein sequence of C12orf40 in vertebrates. The sequences of C12orf40 proteins from vertebrate species were obtained from the NCBI protein database, and the alignment was performed using Multalin (http://multalin.toulouse.inra.fr/multalin/).

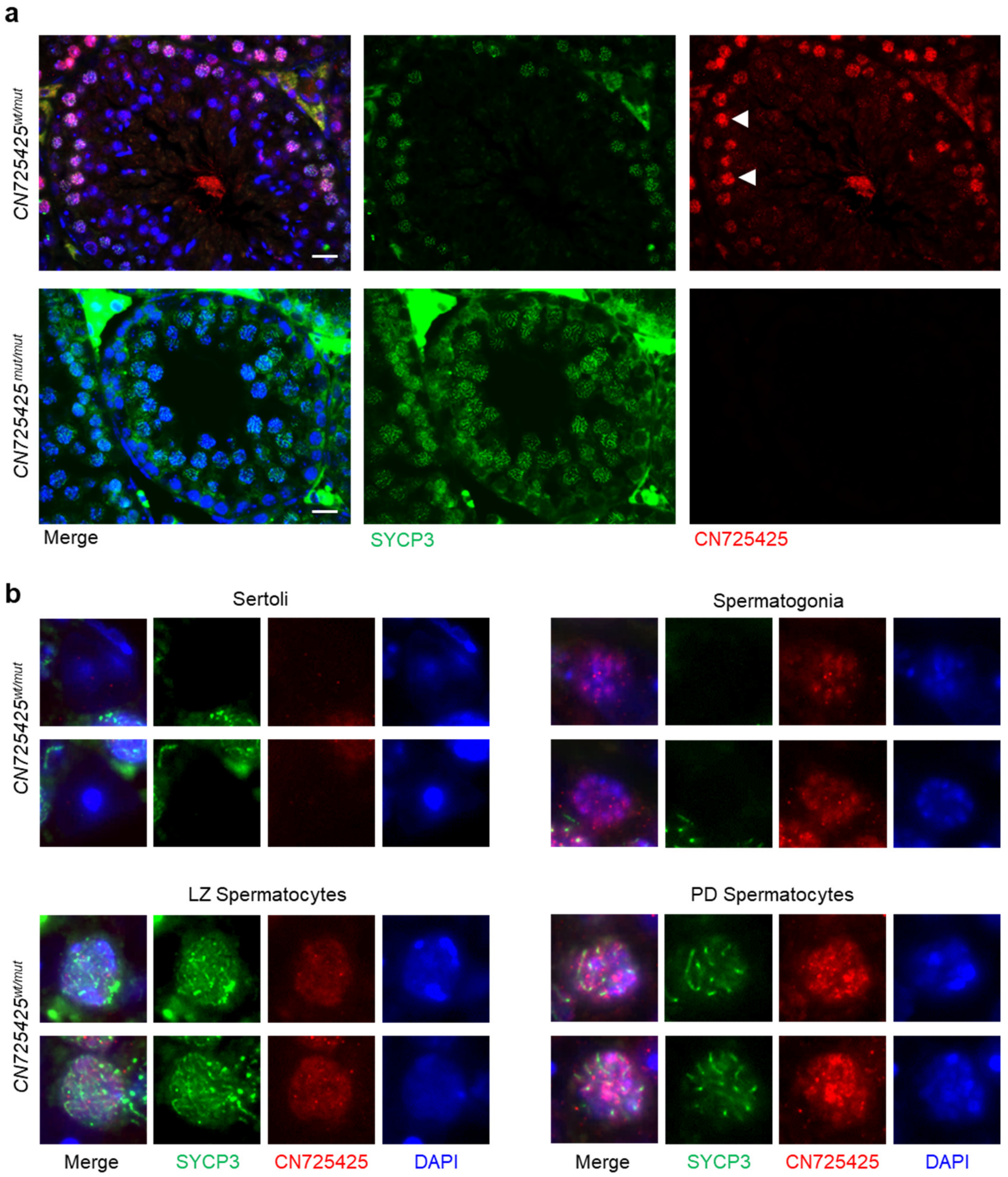

Supplementary Fig. S6 Expression and localization of CN725425 in mouse testes. **a**, **b** Immunostaining showed whole seminiferous tubule (**a**) and enlarged testicular cells (**b**) of testis sections from wildtype and *CN725425*-mutant mice for CN725425 (red) and SYCP3 (green), with nuclei counterstained by DAPI (blue). Scale bar, 50 μm.

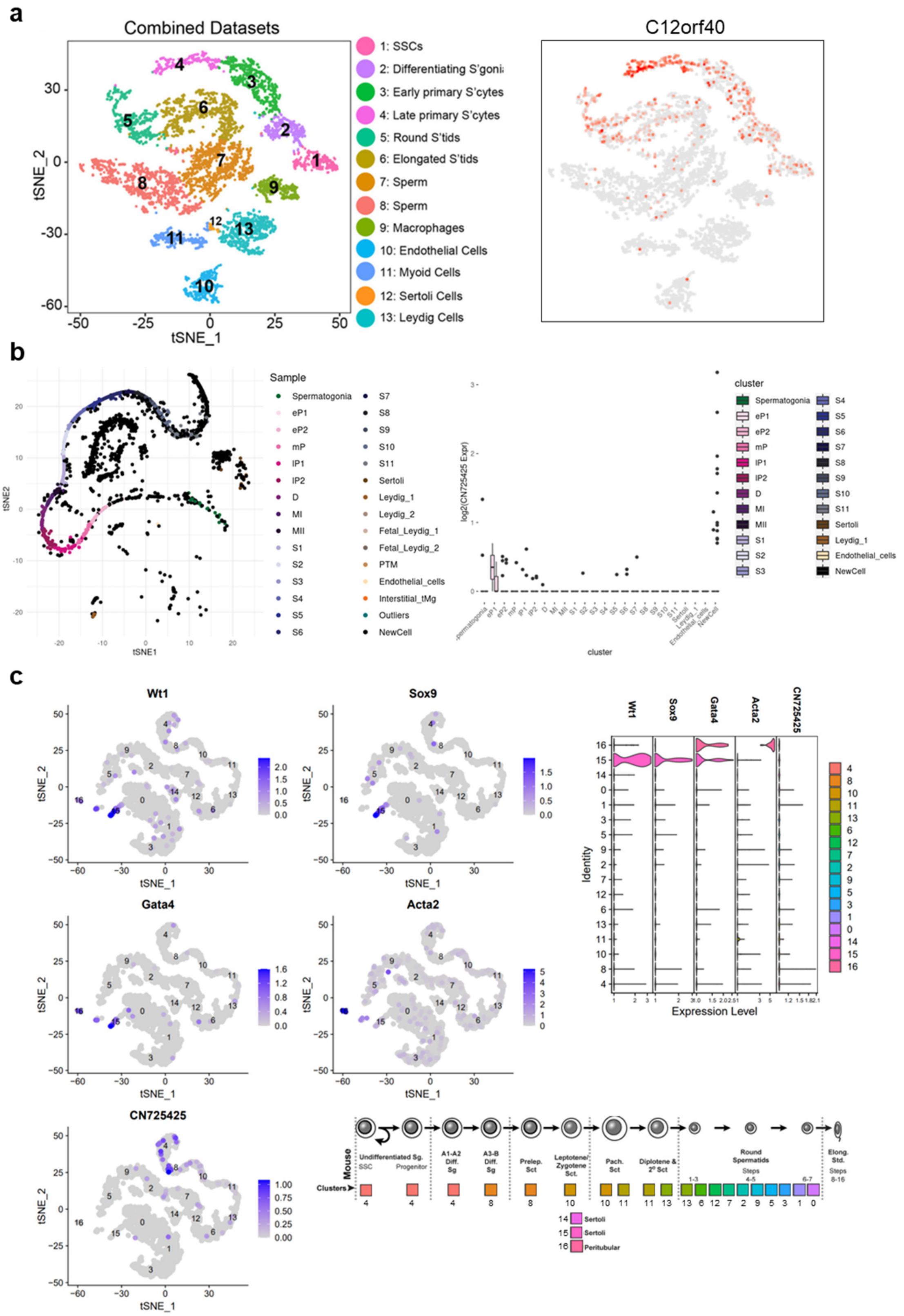

Supplementary Fig. S7 Restricted expression of *C12orf40*/*CN725425* in germ cells.

a Left, tSNE and clustering analysis of combined single-cell transcriptome data from human testes. Right, the expression pattern of *C12orf40* [24]. **b** Left, tSNE and clustering analysis of combined single-cell transcriptome data from mouse testes. Right, boxplot

of the expression pattern of *CN725425* [25]. **c** Left, tSNE and clustering analysis of combined single-cell transcriptome data from mouse testes and the expression of *CN725425* and 4 different somatic cell markers (*Wt1*, *Sox9*, *Gata4* and *Acta2*). Right, violin plot of the expression pattern of *CN725425* and 4 different somatic cell markers (*Wt1*, *Sox9*, *Gata4* and *Acta2*) [26].

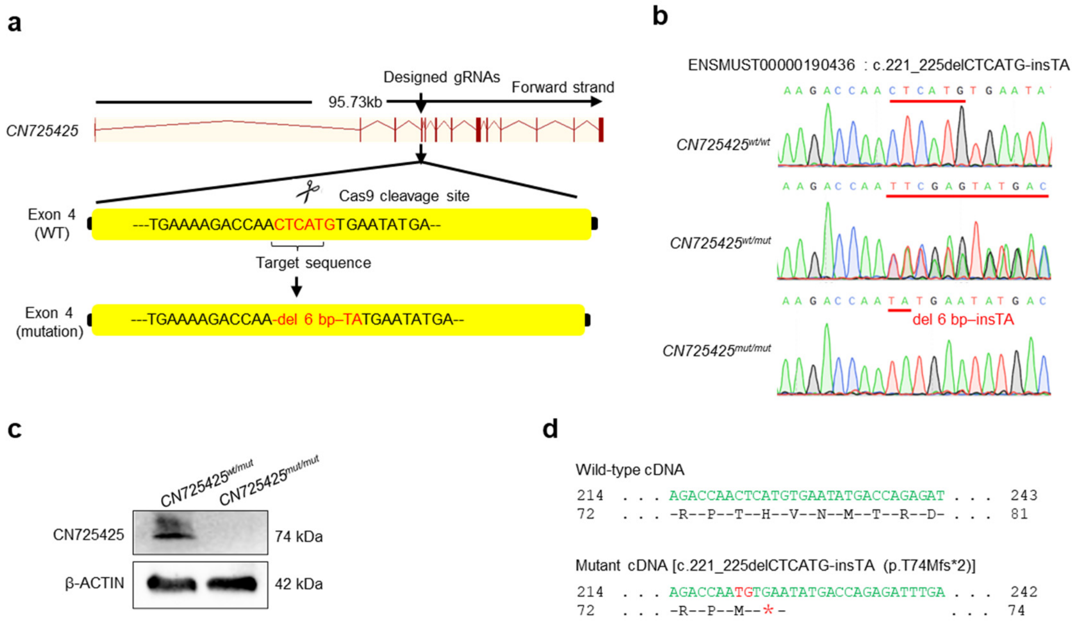

Supplementary Fig. S8 *CN725425*-mutant mice was generated by CRISPR/Cas9. **a** The strategy of *CN725425*-mutant mice generation by CRISPR/Cas9. **b** Chromatogram of the sequences of *CN725425* at targeting region in wildtype, heterozygous, and homozygous mutant mice by Sanger sequencing. A frameshift variant (c.221_225delCTCATG-insTA) at exon 4 of *CN725425* was introduced. **c** Western blotting of CN725425 with testis lysates from two-month-old control and mutant mice, with β-ACTIN serving as a loading control. **d** Functional consequences of the frameshift variant (c.221_225delCTCATG-insTA). The variant leads to frameshift of CN725425 protein (p.T74Mfs*2) from the N terminal.

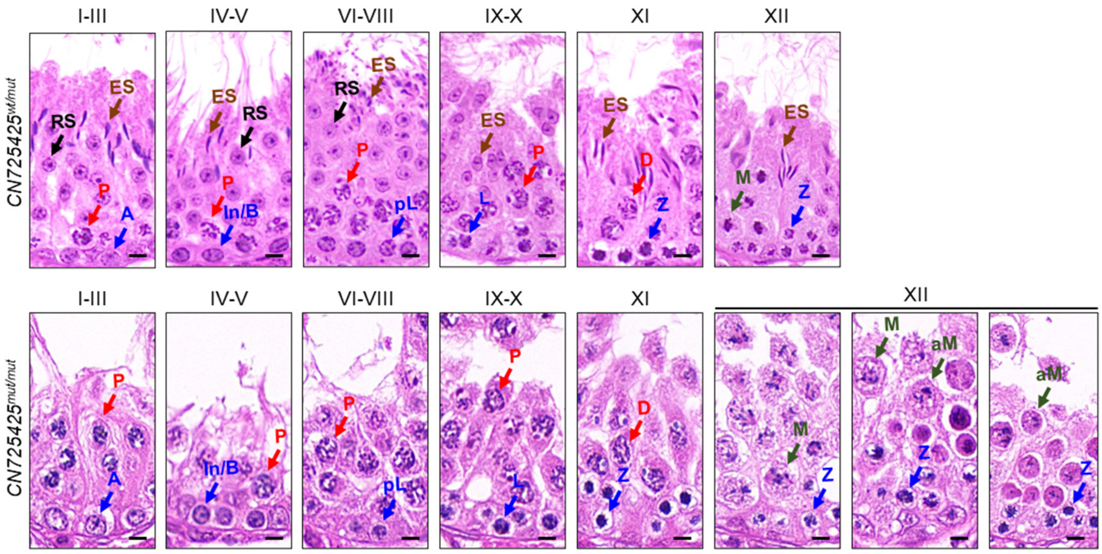

Supplementary Fig. S9 Histological analysis of seminiferous tubules from *CN725425*-mutant mice.

The H&E staining of seminiferous tubules at different stages from control and mutant mice at 2 months of age. A (blue), type A spermatogonia; In (blue), intermediate spermatogonia; B (blue), type B spermatogonia; pL (blue), preleptotene spermatocytes; L (blue), leptotene spermatocytes; Z (blue), zygotene spermatocytes; P (red), pachytene spermatocytes; D (red), diplotene spermatocytes; M (green), meiotic metaphase; aM (green), abnormal meiotic metaphase; RS (black), round spermatids; ES (brown), elongating spermatids. Scale bar, 10 μm.

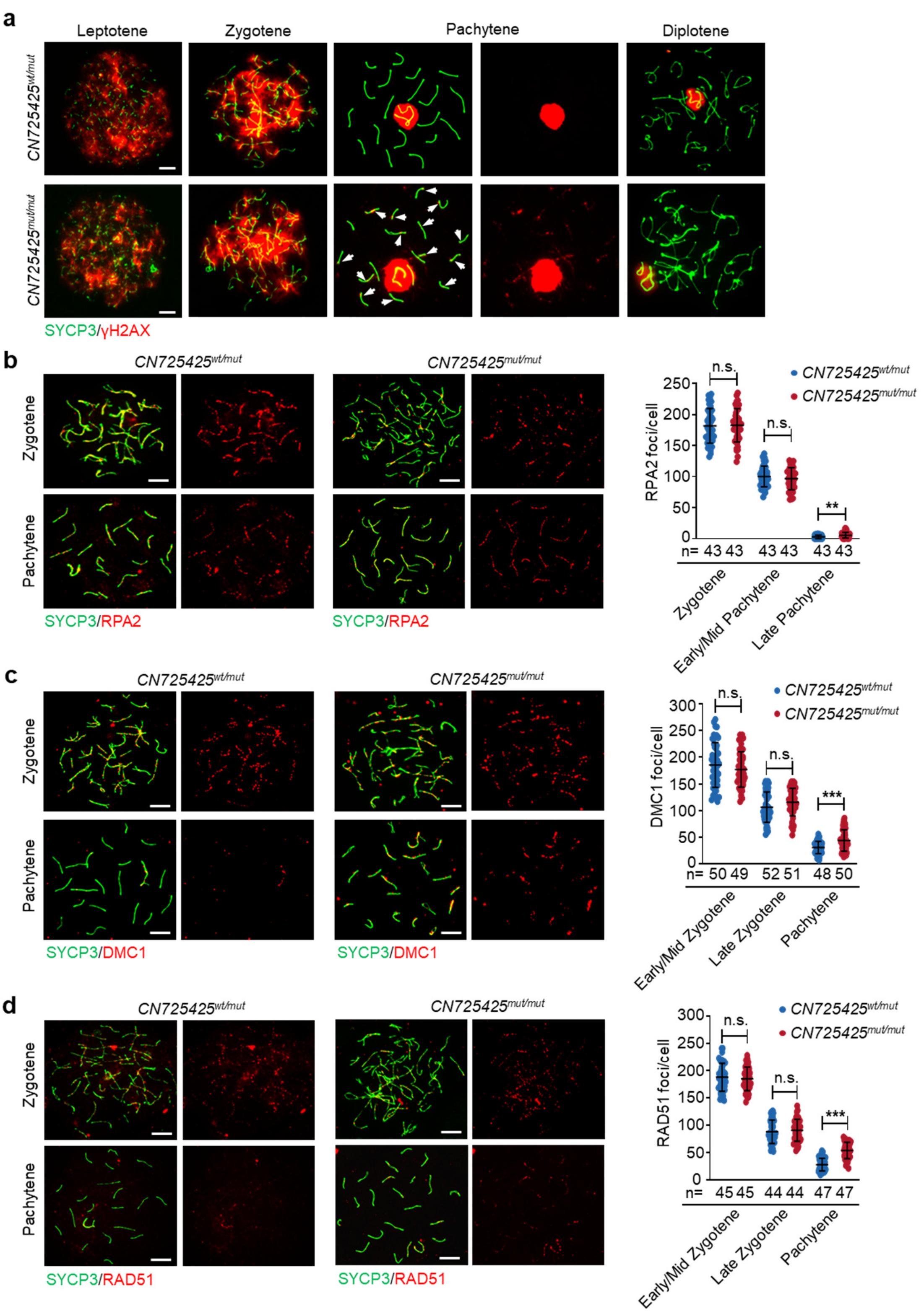

Supplementary Fig. S10 Impaired DSBs repair and homologous recombination in spermatocytes of *CN725425*-mutant mice.

a Representative images of chromosome spreads from control and mutant spermatocytes at indicated stages of meiosis I for SYCP3 (a component of lateral

elements that constitute synaptonemal complex, green) and γH2AX (a general marker of DSBs, red) staining. White arrows indicate hyperaccumulates of γH2AX staining. Scale bar, 10 μm. **b** Left, immunostaining of spermatocyte spreads derived from control and mutant mice for RPA2 (a component of replication protein A complex indicating processed DSBs, red) and SYCP3 (green) at indicated stages. Scale bar, 10 μm. Right, quantification of RPA2 foci in spermatocytes from control and mutant mice at indicated stages (n = 43, 43, 43, 43, 43, and 43, from left to right). **P<0.01. **c** Left, immunostaining of spermatocyte spreads derived from control and mutant mice for DMC1 (DNA strand exchange protein, red) and SYCP3 (green) at indicated stages. Scale bar, 10 μm. Right, quantification of DMC1 foci in spermatocytes from control and mutant mice at indicated stages (n = 50, 49, 52, 51, 48, and 50, from left to right). **P<0.01. **d** Left, immunostaining of spermatocyte spreads derived from control and mutant mice for RAD51 (DNA strand exchange protein, red) and SYCP3 (green) at indicated stages. Scale bar, 10 μm. Right, quantification of RAD51 foci in spermatocytes from control and mutant mice at indicated stages (n = 45, 45, 44, 44, 47, and 47, from left to right). ***P<0.001.

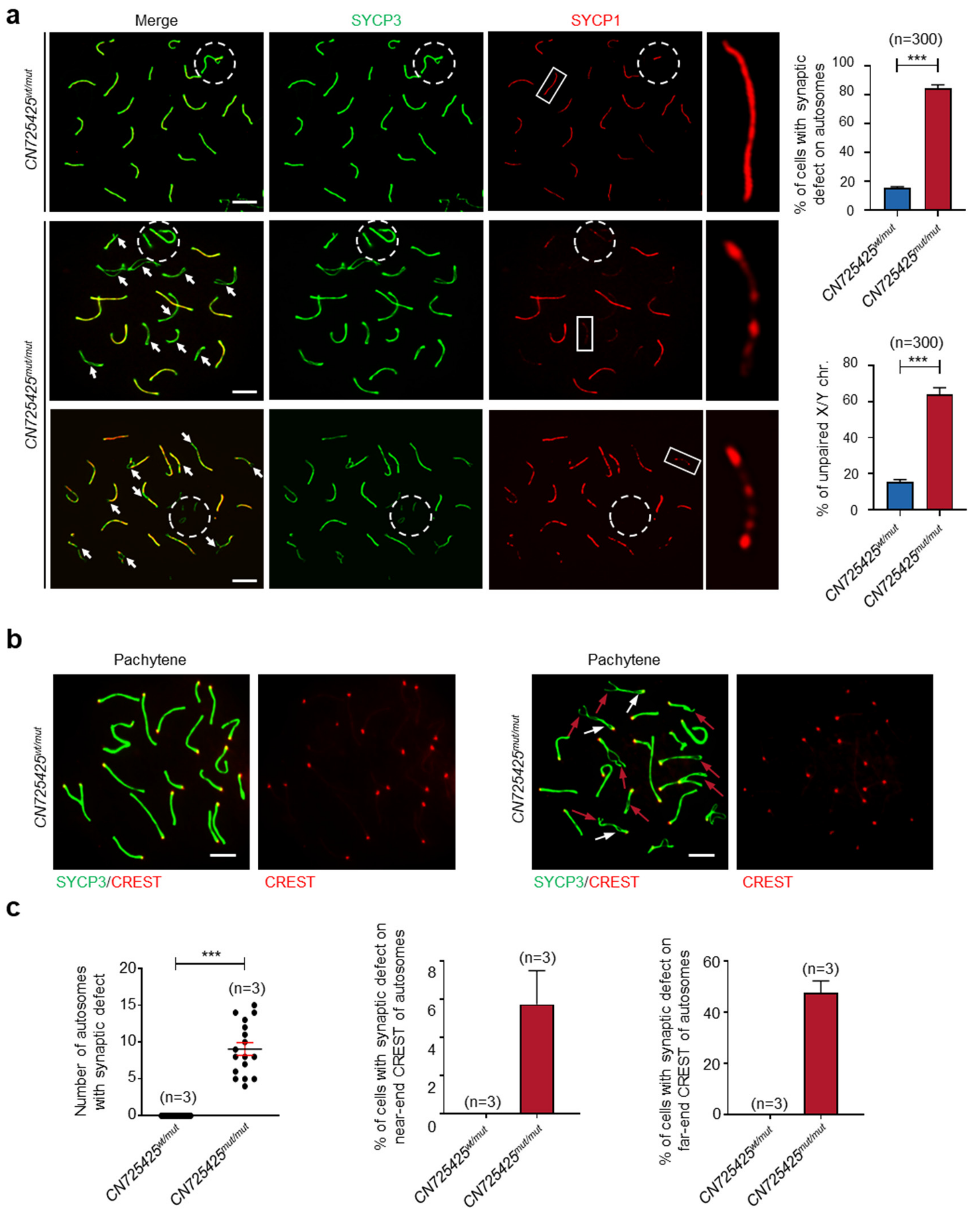

Supplementary Fig. S11 Defective synapsis and failed sex chromosome pairing in response to CN725425 depletion in spermatocytes.

a Left, immunostaining of pachytene spermatocyte spreads derived from control and mutant mice for SYCP1 (a component of both central elements and transverse filaments that constitute synaptonemal complex, red) and SYCP3 (green). The arrows indicate chromosomes with synaptic defects (indicated by the interrupted SYCP1 signals). The dashed circles indicate sex chromosomes. Scale bar, 10 µm. Right, quantification of

pachytene spermatocytes with synaptic defects on autosomes (up panel) and detached sex chromosomes (bottom panel). ***P<0.001. **b** Immunostaining of the chromosome spreads of pachytene spermatocytes from control and mutant mice for CREST (a marker of centromere, red), SYCP3 (green) and nuclei were counterstained with DAPI (blue). White arrows indicate synaptic defect on near-end; red arrows indicate synaptic defect on far-end of centromere region. Scale bar, 20 μm. **c** Quantification of number of autosomes with synaptic defects (left panel), percentage of cell with synaptic defect on near-end (middle panel) or far-end (right panel) of centromere region on autosomes in pachytene spermatocytes from control and mutant mice at indicated stages. ***P<0.001.

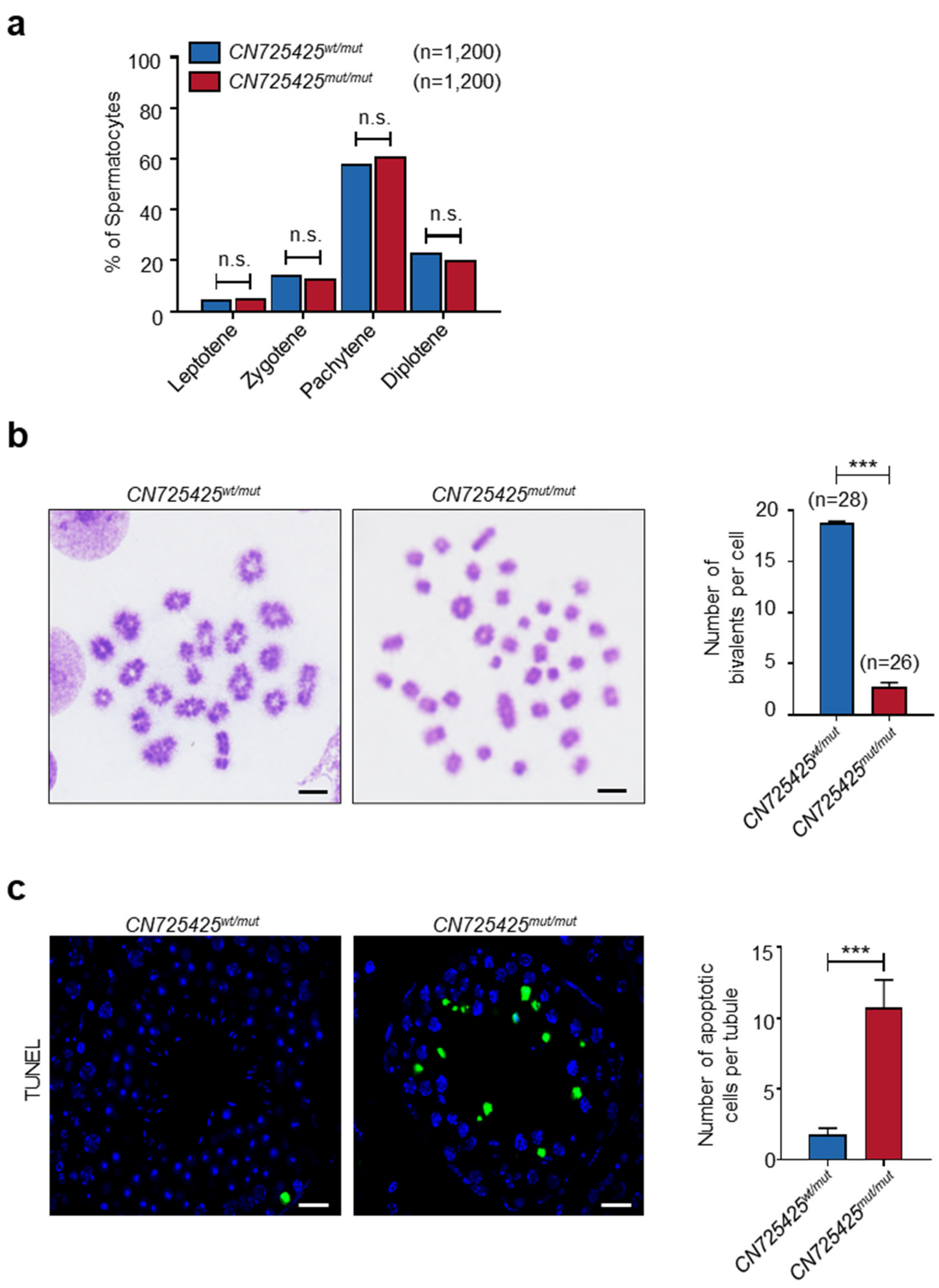

Supplementary Fig. S12 The spermatogenesis of *CN725425*$^{mut/mut}$ males was arrested at MMI.

a Quantification of spermatocytes at indicated meiotic stages from control and mutant males (n=1200 spermatocytes per genotype). **b** Left, Representative images of MMI spermatocytes from control and mutant males stained with Giemsa. Scale bar, 10 µm. Right, quantification of bivalents per spermatocyte. ***P<0.001. **c** Left, TUNEL assay

of testis sections of control and mutant mice at 60-dpp. TUNEL-positive cells (green), nuclei were counterstained with DAPI (blue). Scale bar, 20 µm. Right, quantification of apoptotic germ cells per seminiferous tubule. ***P<0.001.

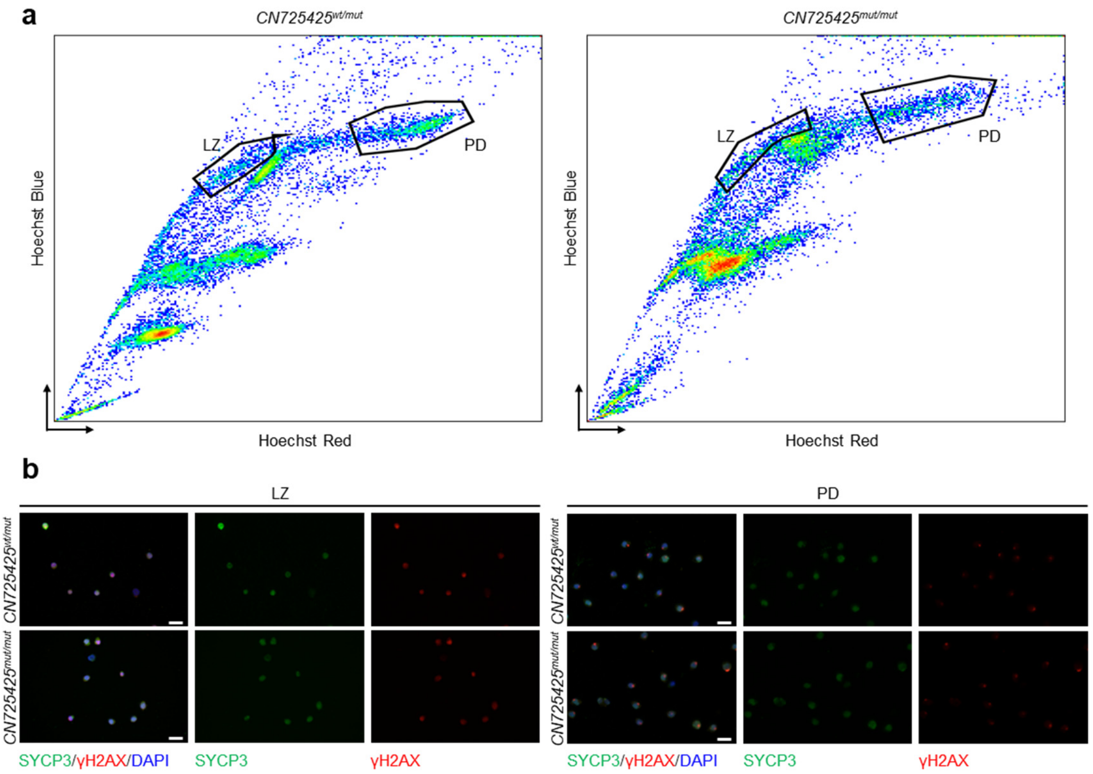

	LZ	PD
CN725425$^{wt/mut}$	82.1%	90.3%
CN725425$^{mut/mut}$	85.7%	92.6%

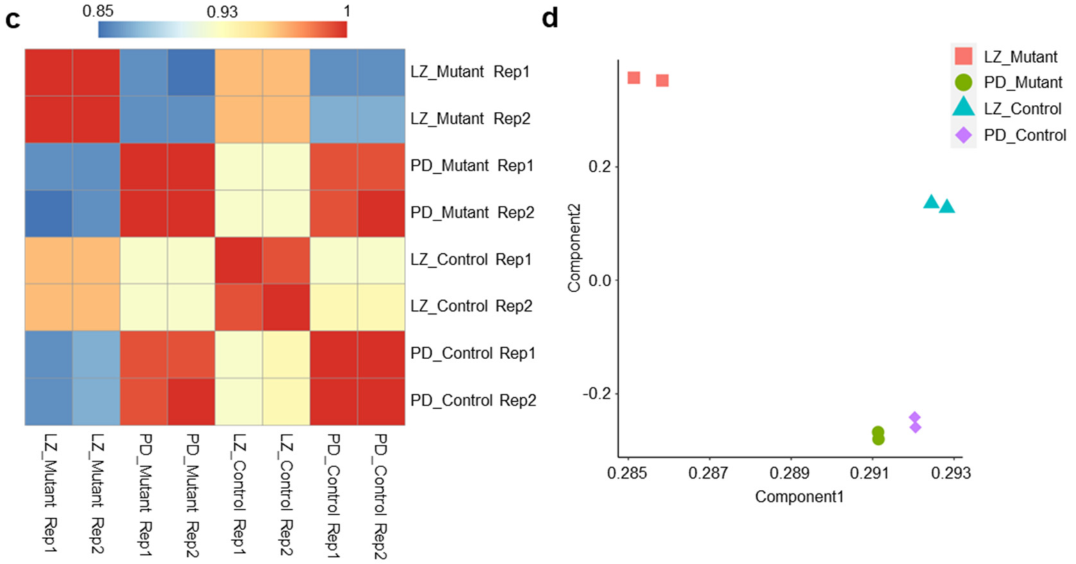

Supplementary Fig. S13 Highly reproducible RNA-seq with isolated control and mutant spermatocytes at LZ and PD stages.

a Flow cytometry analysis of testicular cells from 25-dpp control and mutant mice based on Hoechst fluorescence. **b** Up, Immunostaining of flow cytometry sorted spermatocytes from wildtype and *CN725425*-mutant mice for γH2AX (red) and SYCP3

(green), with nuclei counterstained by DAPI (blue). Scale bar, 20 μm. Bottom, quantification of purity of flow cytometry sorted spermatocytes in each group (n>250). **c**, **d** The reproducibility analysis of RNA-seq data by Pearson correlation (**c**) and PCA (**d**) analysis.

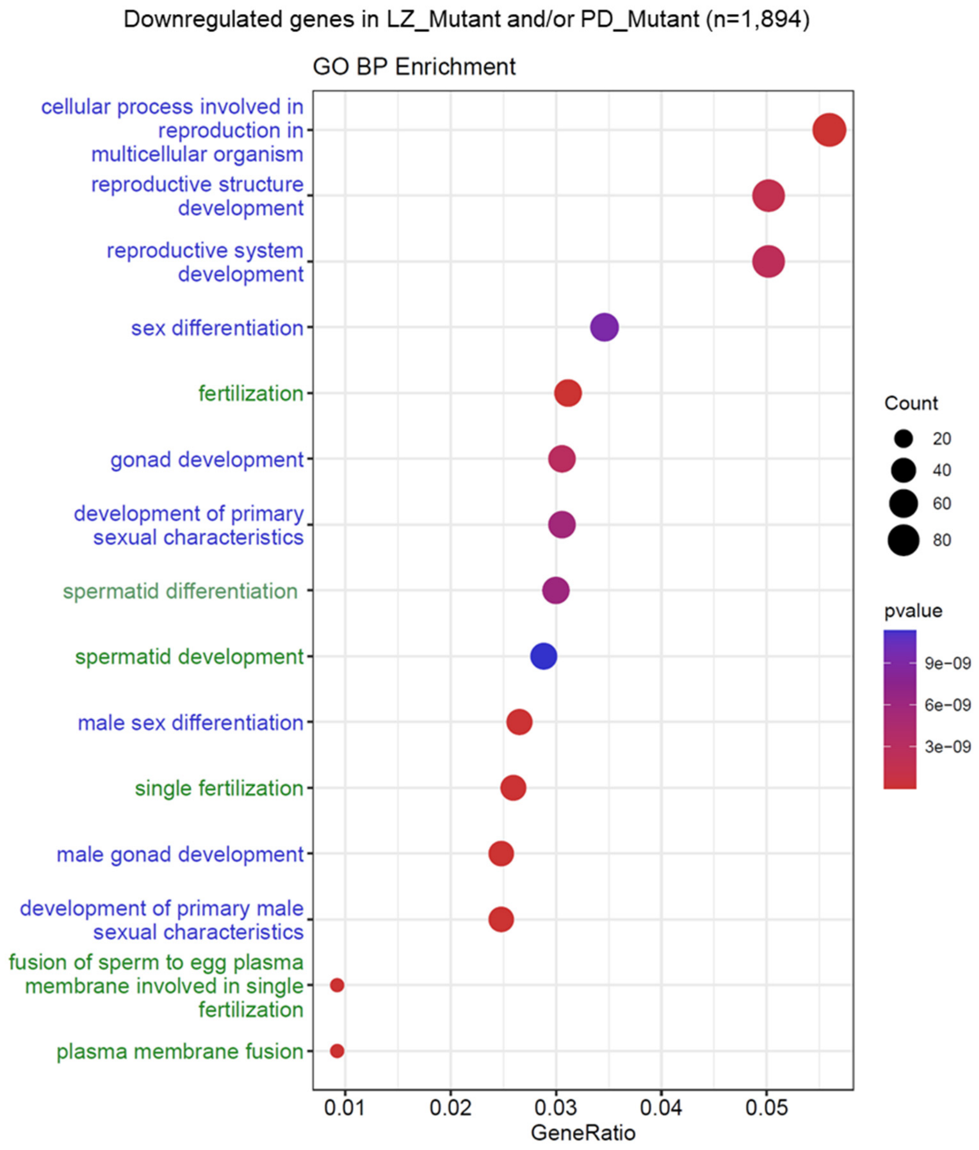

Supplementary Fig. S14 Gene Ontology analysis of downregulated genes in response to CN725425 depletion.

Dotplot of Gene Ontology Biological Process (GO BP) terms overrepresented by downregulated genes in mutant spermatocytes at LZ and/or PD stage. General germ cell development-related GO categories are highlighted in blue. Spermiogenesis-related GO categories are highlighted in green.

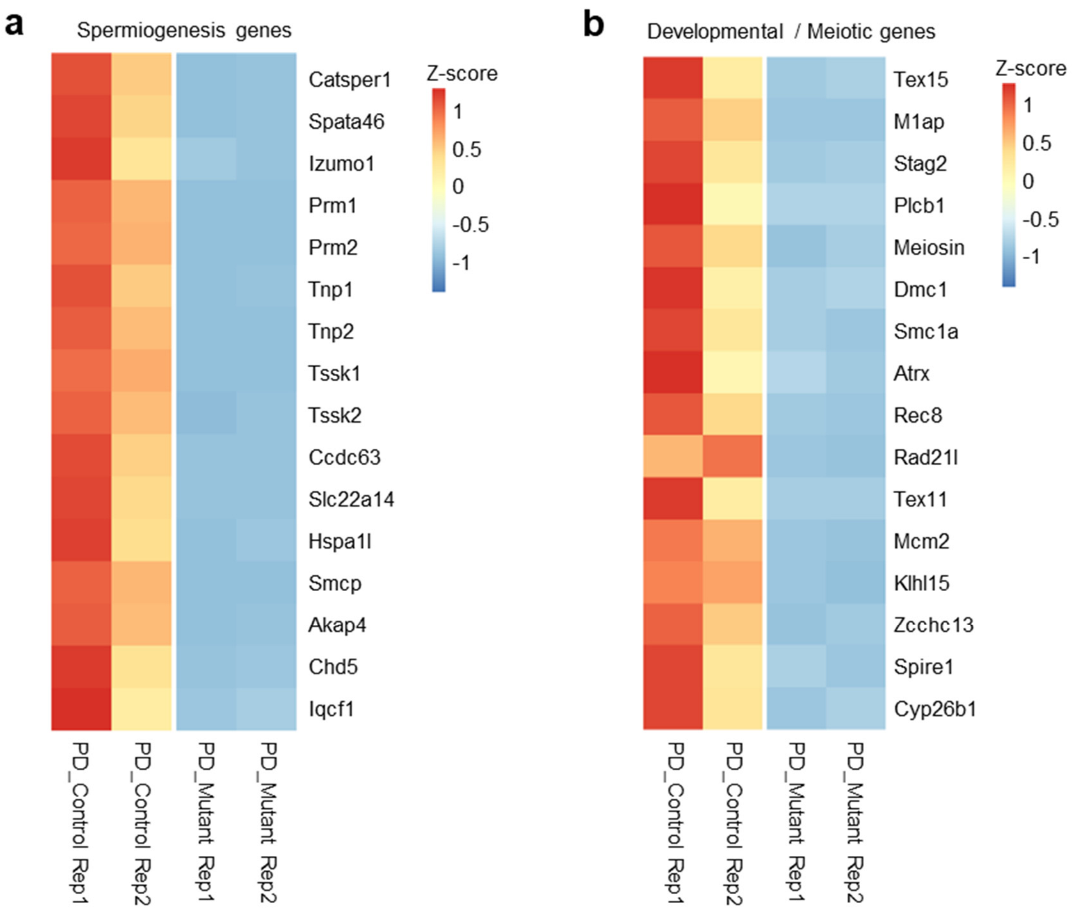

Supplementary Fig. S15 Downregulated genes in *CN725425*-depleted spermatocytes are enriched in spermiogenesis or general germ cell development. **a**, **b** heatmap of expression change of selected spermiogenesis genes (**a**) and developmental/meiotic genes (**b**) in mutant PD spermatocytes.

a

Biological process predictions of CN725425 by FFPred Predictions

GO term	Name	Prob	SVM Reliability
GO:2001141	regulation of RNA biosynthetic process	0.930	H
GO:0019222	regulation of metabolic process	0.923	H
GO:0034645	cellular macromolecule biosynthetic process	0.909	H
GO:1903506	regulation of nucleic acid-templated transcription	0.909	H
GO:0010468	regulation of gene expression	0.897	H
GO:0051171	regulation of nitrogen compound metabolic process	0.889	H
GO:0006351	transcription, DNA-templated	0.877	H
GO:0006355	regulation of transcription, DNA-templated	0.869	H
GO:0051252	regulation of RNA metabolic process	0.852	H
GO:0009059	macromolecule biosynthetic process	0.834	H
GO:0000375	RNA splicing, via transesterification reactions	0.750	H
GO:0010629	negative regulation of gene expression	0.750	H
GO:0006357	regulation of transcription from RNA polymerase II promoter	0.746	H
GO:0000398	mRNA splicing, via spliceosome	0.733	H
GO:0016071	mRNA metabolic process	0.693	H
GO:0008380	RNA splicing	0.686	H
GO:0006366	transcription from RNA polymerase II promoter	0.620	H
GO:0010628	positive regulation of gene expression	0.608	H
GO:0031328	positive regulation of cellular biosynthetic process	0.593	H
GO:1903507	negative regulation of nucleic acid-templated transcription	0.588	H
GO:0010557	positive regulation of macromolecule biosynthetic process	0.587	H
GO:0045944	positive regulation of transcription from RNA polymerase II promoter	0.580	H
GO:0009890	negative regulation of biosynthetic process	0.575	H
GO:0006810	transport	0.540	H
GO:0019538	protein metabolic process	0.558	L
GO:0016070	RNA metabolic process	0.779	L

Molecular function predictions of CN725425 by FFPred Predictions

GO term	Name	Prob	SVM Reliability
GO:0003676	nucleic acid binding	0.979	H
GO:0003677	DNA binding	0.921	H
GO:0000981	sequence-specific DNA binding RNA polymerase II transcription factor activity	0.888	H
GO:0001071	nucleic acid binding transcription factor activity	0.885	H
GO:0003700	sequence-specific DNA binding transcription factor activity	0.868	H
GO:0001228	RNA polymerase II transcription regulatory region sequence-specific DNA binding transcription factor activity involved in positive regulation of transcription	0.854	H
GO:0003723	RNA binding	0.761	H
GO:0001077	RNA polymerase II core promoter proximal region sequence-specific DNA binding transcription factor activity involved in positive regulation of transcription	0.698	H
GO:0043565	sequence-specific DNA binding	0.656	H
GO:0008092	cytoskeletal protein binding	0.640	H
GO:0046982	protein heterodimerization activity	0.616	H
GO:0019900	kinase binding	0.567	H
GO:0000975	regulatory region DNA binding	0.560	H
GO:0044822	poly(A) RNA binding	0.556	H
GO:0097159	organic cyclic compound binding	0.978	L
GO:0046872	metal ion binding	0.770	L
GO:0008134	transcription factor binding	0.726	L
GO:0005102	receptor binding	0.714	L
GO:0032403	protein complex binding	0.707	L
GO:0043169	cation binding	0.665	L
GO:0036094	small molecule binding	0.656	L
GO:0019904	protein domain specific binding	0.546	L

b

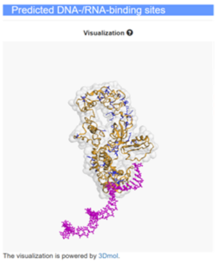

DNA binding

PDB template	Ligand	Download	Sequence length	Number of predicted binding sites
2bsqF	DNA	Complex	652	47

Predicted binding residues

6,7,8,10,12,17,18,44,45,46,48,64,65,72,77,83,84,99,121,133,136,212,225,264,266,334,337,339,377,416,417,420,457,460,461,469,471,474,476,489,508,509,538,569,581,614,615

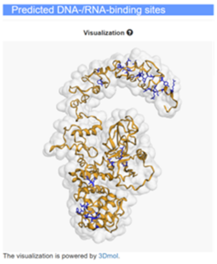

RNA binding

PDB template	Ligand	Download	Sequence length	Number of predicted binding sites
N/A	N/A	N/A	652	35

Predicted binding residues

10,15,17,18,21,23,27,28,31,32,61,64,65,75,84,86,87,114,117,364,367,368,369,370,371,373,374,398,412,462,469,470,581,583,637

Supplementary Fig. S16 Prediction of nucleic acid binding ability of CN725425.

a Predicted GO term related to CN725425 by FFPred [27]. **b** Prediction output of DNA- and RNA-binding site of CN725425 by NucBind [28].

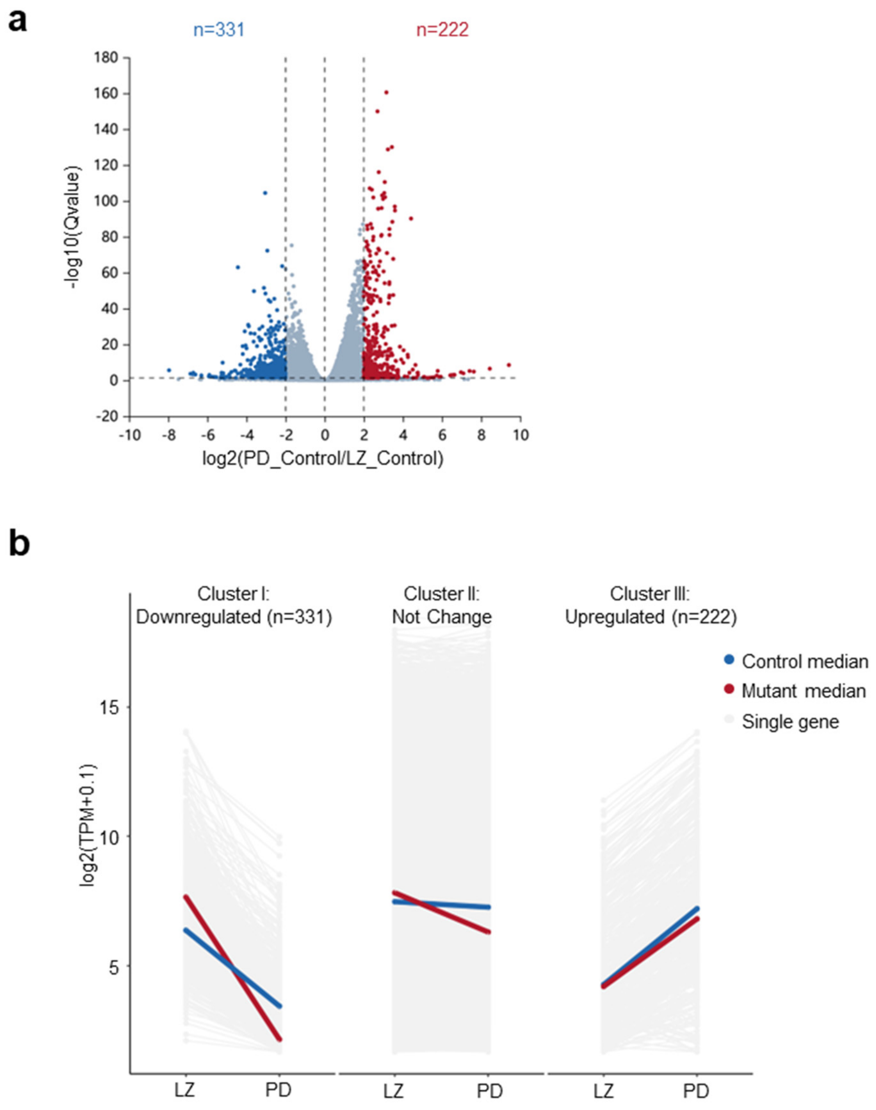

Supplementary Fig. S17 Dynamic changes of transcriptomic profile during LZ-to-PD transition.

a Volcano plots showing up- (red) and downregulated (blue) genes during LZ-to-PD transition in control testes. **b** Three classified gene clusters by gene expression pattern during control LZ-to-PD transition (left, downregulated; middle, unchanged; right, upregulated). Each grey line represents the expression of a single gene, the blue and red line represent the median expression of the cluster during control and mutant LZ-to-PD transition, respectively.

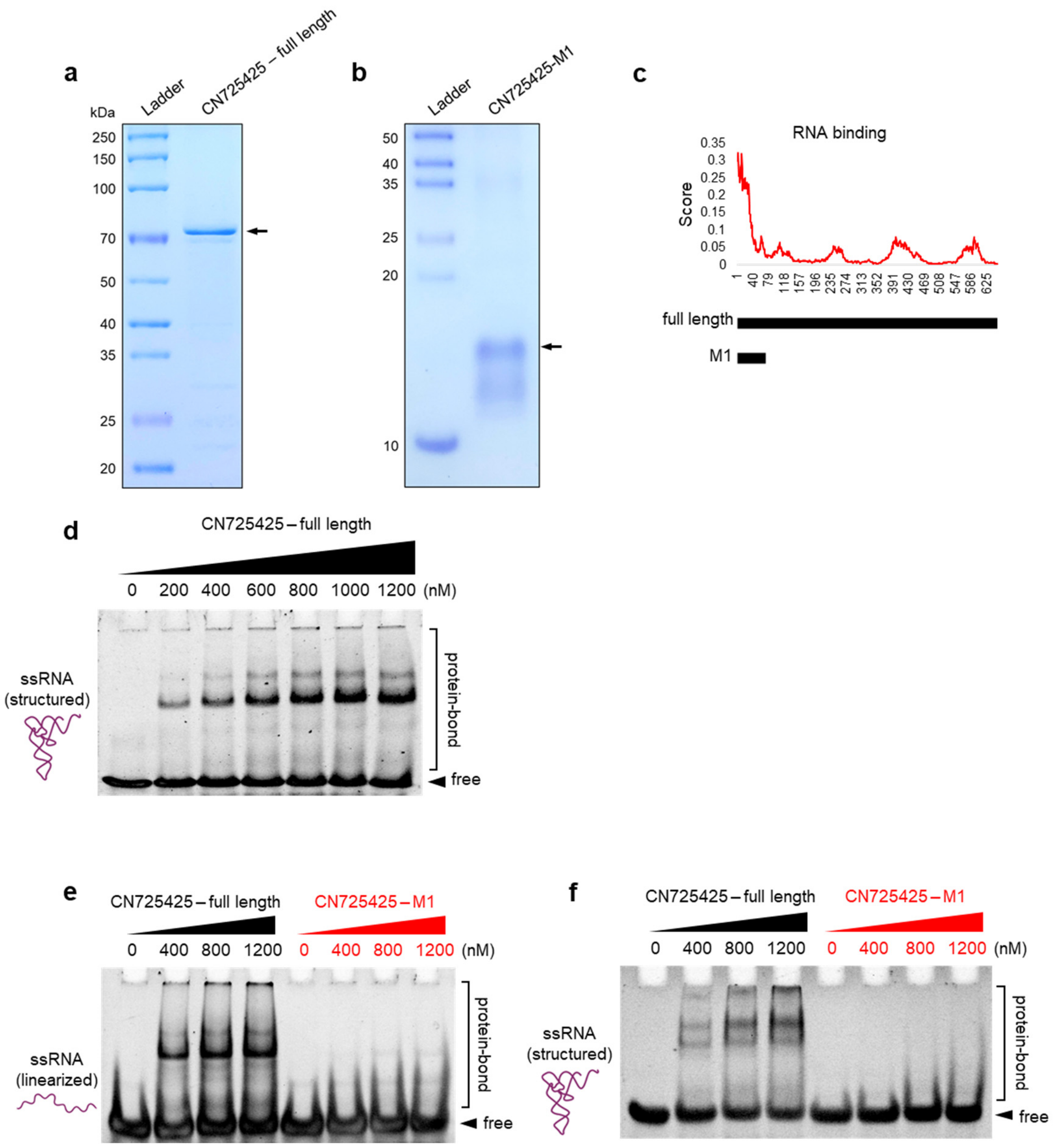

Supplementary Fig. S18 RNA-binding capacity test of CN725425 by Electrophoretic mobility shift assay.

a, **b** Recombinant full length (**a**) and M1 mutant (**b**) CN725425 used in this study. The 8% (**a**) and 15% (**b**) SDS–polyacrylamide gel was stained with Coomassie blue. Arrows indicate specific bands. **c** Schematics of predicted CN725425 RNA binding profile by DisoRDPbind and length for CN725425 truncations [29]. **d-f** Electrophoretic mobility shift assays with CN725425 and indicated RNA substrates. The experiments were performed three times.

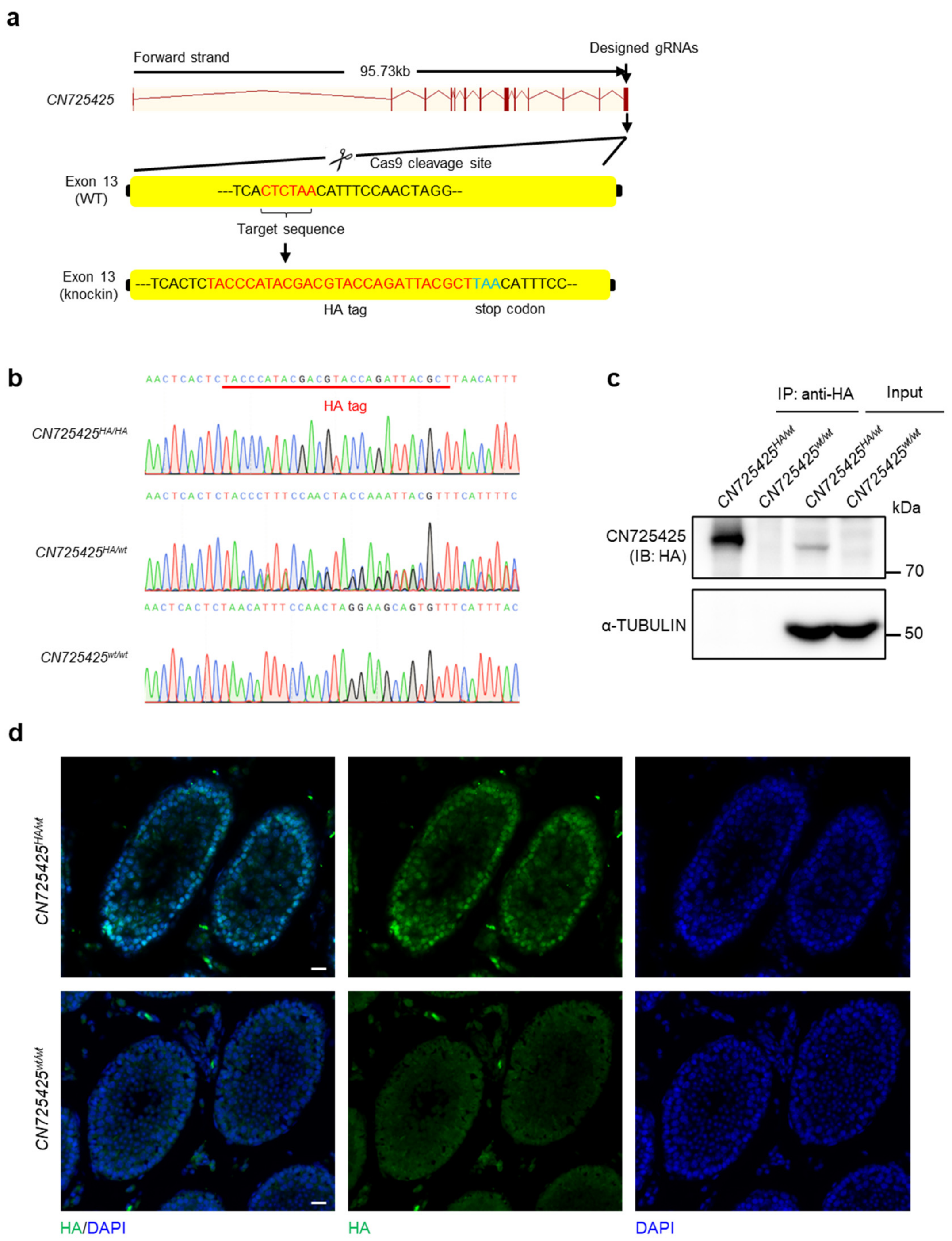

Supplementary Fig. S19 ***CN725425-HA*** **tagged mice was generated by CRISPR/Cas9.**

a The strategy of *CN725425-HA* knock-in mice generation by CRISPR/Cas9. **b** Chromatogram of the sequences of *CN725425* at targeting region in wildtype, heterozygous, and homozygous knock-in mice by Sanger sequencing. **c** Western blotting of CN725425-HA with testis lysates immunoprecipitated by anti-HA antibody and total testis lysates from 25-dpp control and knock-in mice, with α-TUBULIN

serving as a loading control. **d** Immunostaining showed whole seminiferous tubules of testis sections from CN725425-HA mice for CN725425 (green), with nuclei counterstained by DAPI (blue). Scale bar, 50 μm.

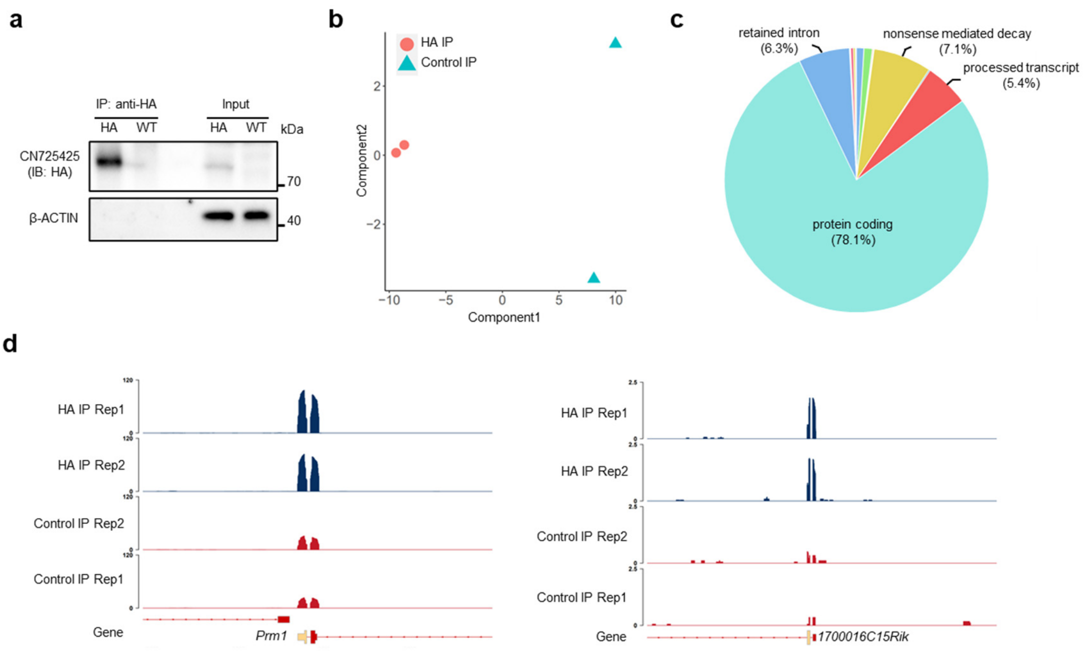

Supplementary Fig. S20 Identification of CN725425-associated RNAs by RIP-Seq. **a** Western blotting of CN725425-HA with testis lysates immunoprecipitated by anti-HA antibody and total testis lysates from 18-dpp control and CN725425-HA tag mice, with β-ACTIN serving as a loading control. **b** PCA analysis of RIP-seq data using CN725425-HA tag mouse testis. **c** Pie chart of HA enriched regions. **d** Track view of selected HA enriched regions. The left panel shows HA enrichment at the 3'UTR of Prm1, and the right panel indicates HA enrichment at 3'UTR of 1700016C15Rik (mouse ortholog of human SPMIP3, sperm associated microtubule inner protein 3).

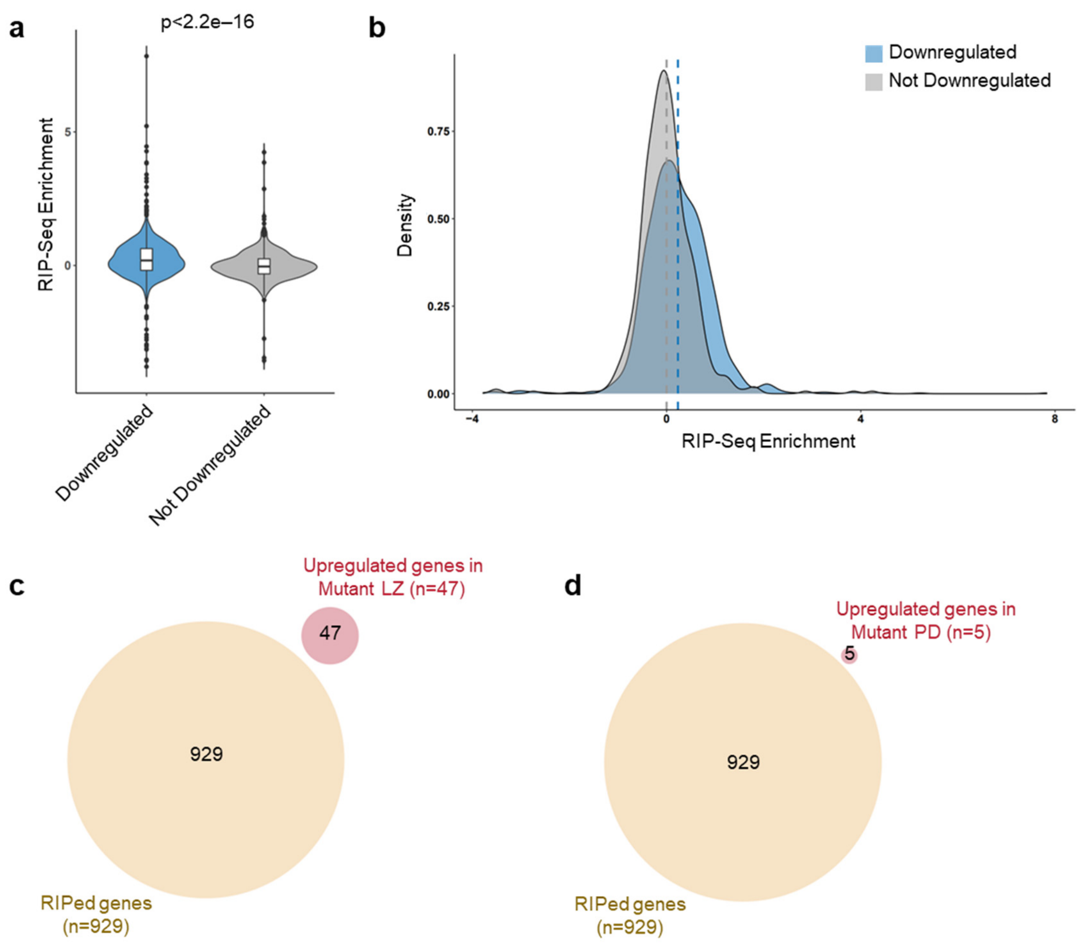

Supplementary Fig. S21 Stabilizing role of CN725425 on its binding RNAs.

a Violin plot of RIP-seq enrichment score (i.e. Fold change between HA and Control IP signal) of downregulated genes and not downregulated genes between Mutant and Control PD. P value was calculated by Two-Samples Wilcoxon Test. **b** Density plot of RIP-seq enrichment score (i.e. Fold change between HA and Control IP signal) of downregulated genes and not downregulated genes between Mutant and Control PD. Dotted line indicates the mean value. **c** Venn diagram showing shared and unique genes between CN725425-RIPed mRNAs and upregulated genes between Mutant and Control spermatocytes at LZ stage. **d** Venn diagram showing shared and unique genes between CN725425-RIPed mRNAs and upregulated genes between Mutant and Control spermatocytes at PD stage.

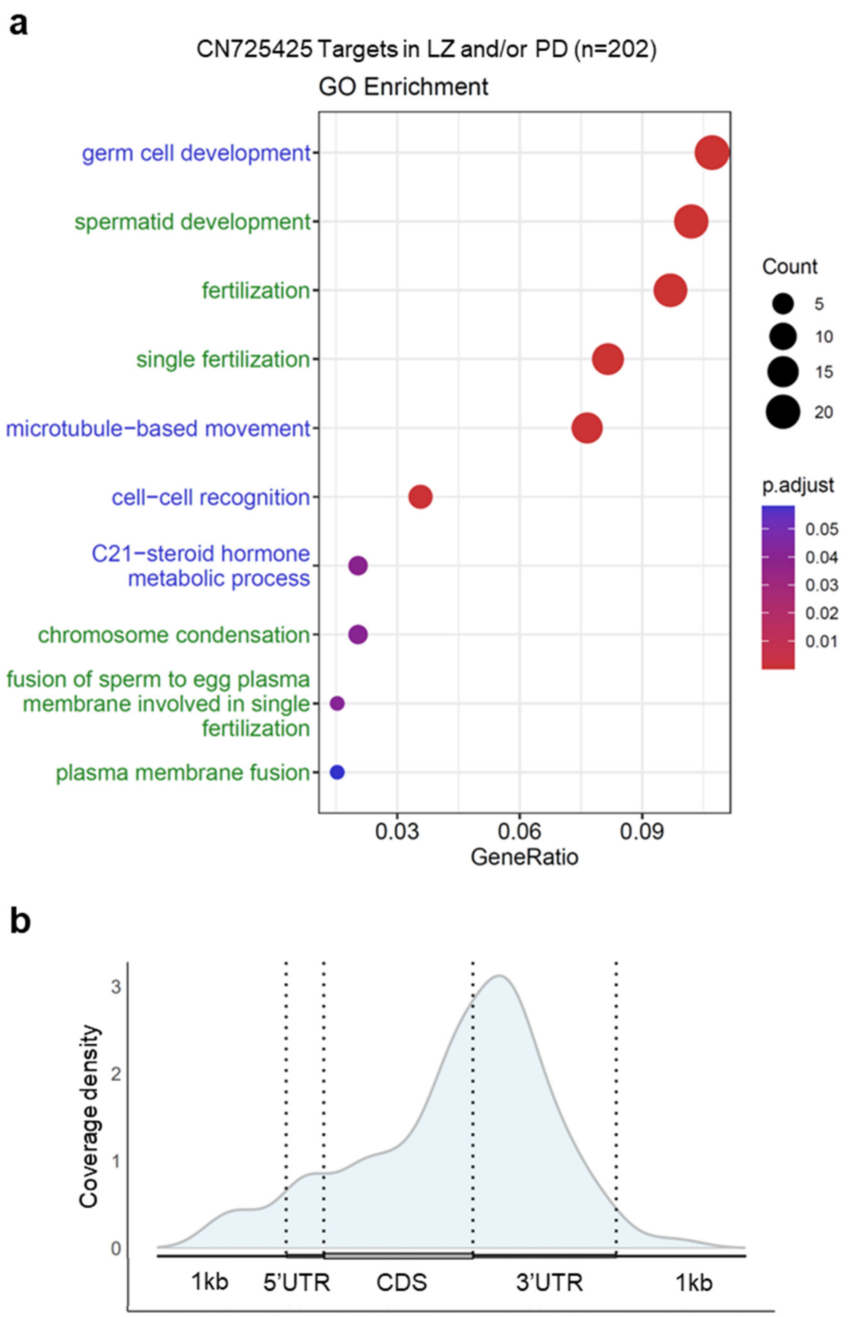

Supplementary Fig. S22 Features of direct regulatory targets by CN725425.

a GO enrichment analysis of 202 target genes of CN725425. General germ cell development-related GO categories are highlighted in blue. Spermiogenesis-related GO categories are highlighted in green. **b** Metagene analysis of HA enriched regions in 202 target genes of CN725425.

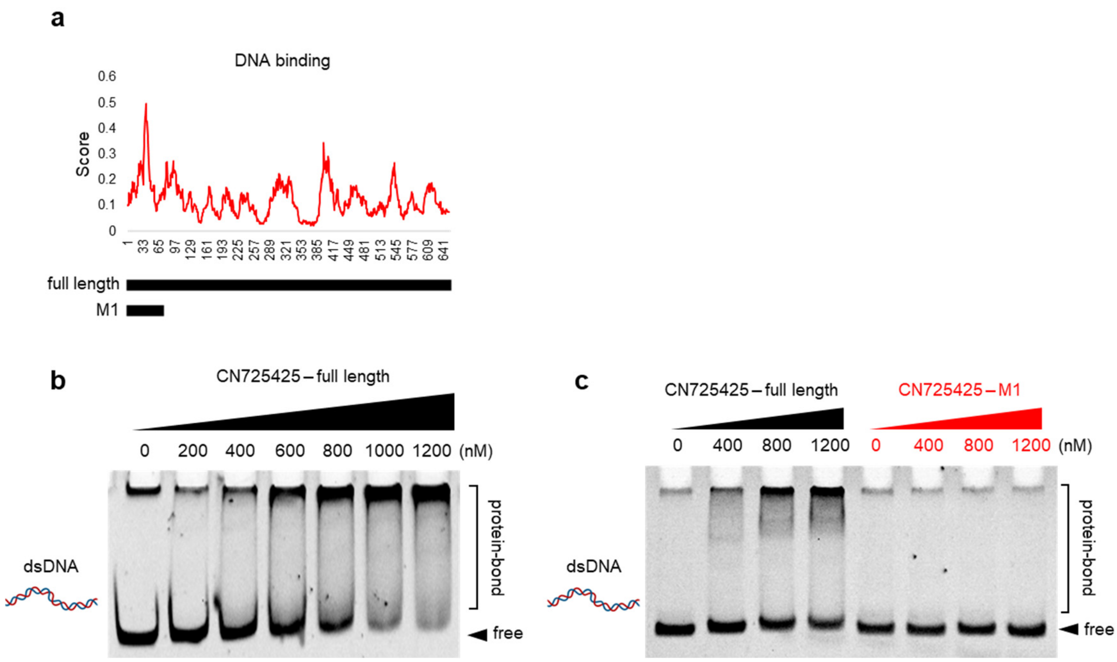

Supplementary Fig. S23 DNA-binding ability test of CN725425 by Electrophoretic mobility shift assay.

a Schematics of predicted CN725425 DNA binding profile by DisoRDPbind and length for CN725425 truncations [29]. **b**, **c** Electrophoretic mobility shift assays with CN725425 and indicated DNA substrates. The experiments were performed three times.

Supplementary tables

Supplementary Table S1. Homozygous variants of *C12orf40* identified in subjects with NOA.

	**T00166 II-1, Y9770 II-1**	**T00579 II-1, II-2**
cDNA alteration[a]	c.232_233insTT	c.1286+1G>A
Protein alteration	p.M78Ifs*2	-
Variant type	frameshift	splicing site
Variant allele	homozygous	homozygous
1000 Genomes Project	0	0
East Asians in gnomAD	0.0019	0
All individuals in gnomAD	0.00009771	0.00002442
SIFT	NA	NA
PolyPhen-2	NA	NA
MutationTaster	NA	damaging
CADD[b]	NA	24.3

Abbreviation: [a] NCBI reference sequence number of *C12orf40* is NM_001031748.4.

[b] Variants with CADD values greater than 4 are considered to be deleterious. NA, not available.

Supplementary Table S2. Semen routine parameters in men with *C12orf40* variants.

	T00166 II-1	Y9770 II-1	T00579 II-1	T00579 II-2	Reference Limits
Age	32	32	37	31	-
Origin	Chinese	Chinese	Chinese	Chinese	-
Male karyotype	46, XY	46, XY	46, XY	46, XY	-
Y-Chromosome	No deletion	No deletion	No deletion	No deletion	-
**Semen analysis** [a]					
Semen volume (mL)	3.3	4.4	2.7	4.1	1.5
Sperm concentration (10^6/mL)	0	0	0	0	15.0
**Hormone analysis** [b]					
FSH (mIU/mL)	3.29	4.19	9.44	NA	M: 0.95–11.95<br>F: 1.38–133.41
Testosterone (ng/mL)	6.95	5.25	7.33	NA	1.42–9.23
LH (mIU/mL)	2.76	2.64	4.45	NA	M: 0.57-12.07<br>F: 1.80–89.08

Abbreviation: [a] Reference limits according to the WHO (2010) standards.[30]

[b] Reference values were suggested by the local clinical laboratory. NA, not available.

Supplementary Table S3. A list of primers used in this study.

Primer name	F/R	Primer Sequence (5' to 3')	Annealing Temperature (°C)	Length (bp)
C12orf40 (c.232_233insT)	F1	AATAGGGCAGCTATAATGAGGAATA	57	465
	R1	AACAAAAACTGGGGGTAGAAGGT		
C12orf40 (c.1286+1G>A)	F2	ACCAGTGGTTTTCCTCTGTACG	57	281
	R2	CACCCTCAAATGGCAATACGC		
C12orf40 (E8-E10)	F4	CCAGAAAAATGTCAGCCAAACAAG	56.5	237
	R4	TCCAAAGTCCCATGACTGCTTA		
C12orf40 (RT-PCR in Mice)	F1	CTGGGTGTTGTTTCACCTGTC	57	398
	R1	GTGGAATGTTGGCTCCGAAT		
Actin (RT-PCR in Mice)	F1	AGATCAAGATCATTGCTCCTCC	57	170
	R1	AGCTCAGTAACAGTCCGCCT		
C12orf40-KO (Genotype identification)	F1	TTAGAGCCCTTTAATTGAAGCAGG	59	442
	R1	GAATGACATCAACAAGCAGTGGAG		
C12orf40-KI (Genotype identification)	F1	GCCACAGAGACAACTGGTAAT	58	713
	R1	GTTCCATCCTGACTTCCTTTGA		

Abbreviation: F, forward primers; R, reverse primers; KO, knock-out.

Supplementary Table S4. A list of antibodies used in the study.

Name of antibodies	Catalog number	Host species	Source	Application
CN725425	-	Rabbit	Made by Sinobiological	WB (1:1000)
CN725425	-	Rabbit	Made by Abclonal	IF (1:30)
SYCP3	ab205846	Mouse	Abcam	IF (1:400)
SYCP3	ab15093	Rabbit	Abcam	IF (1:100)
SYCP1	ab15090	Rabbit	Abcam	IF (1:200)
γ-H2AX	ab11174	Rabbit	Abcam	IF (1:1000)
MLH1	551092	Mouse	Bdbiosciences	IF (1:30)
DMC1	-	Rabbit	Gift from PhD. Mengcheng Luo	IF (1:50)
RAD51	-	Rabbit	Gift from PhD. Mengcheng Luo	IF (1:50)
RPA2	-	Rabbit	Gift from PhD. Mengcheng Luo	IF (1:50)
CREST	Immunovision	Human	HCT-0100	IF (1:50)
PH3	AP0002	Rabbit	Abclonal	IF (1:100)
H1t	A18597	Rabbit	ABclonal	IF (1:100)
HA-Tag	3724	Rabbit	CST	WB (1:2000)<br>IF (1:250)<br>IP (1:100)
Peanut agglutinin (PNA), rhodamine	RL-1072	-	Vector Laboratories	IF (1:1000)
Alexa Fluor 555	A-31572	Donkey	Invitrogen	IF (1:500)
Alexa Fluor 488	A-21121	Goat	Invitrogen	IF (1:500)
ACTIN	AB0011	Mouse	Abways	WB (1:2000)
GAPDH	AB0036	Mouse	Abways	WB (1:2000)
HRP-conjugated Anti-mouse IgG	GAM007-100	Goat	MultiSciences	WB (1:5000)
HRP-conjugated Anti-rabbit IgG	GAR007-100	Goat	MultiSciences	WB (1:5000)
